# Ercc1 DNA repair deficiency results in vascular aging characterized by VSMC phenotype switching, ECM remodeling, and an increased stress response

**DOI:** 10.1111/acel.14126

**Published:** 2024-03-07

**Authors:** Janette van der Linden, Sanne J. M. Stefens, José María Heredia‐Genestar, Yanto Ridwan, Renata M. C. Brandt, Nicole van Vliet, Isa de Beer, Bibi S. van Thiel, Herman Steen, Caroline Cheng, Anton J. M. Roks, A. H. Jan Danser, Jeroen Essers, Ingrid van der Pluijm

**Affiliations:** ^1^ Division of Vascular Medicine and Pharmacology, Department of Internal Medicine Erasmus University Medical Center Rotterdam The Netherlands; ^2^ Department of Molecular Genetics, Cancer Genomics Center Erasmus University Medical Center Rotterdam The Netherlands; ^3^ AMIE Core facility Erasmus University Medical Center Rotterdam The Netherlands; ^4^ Cortalix BV Groningen The Netherlands; ^5^ Division of Experimental Cardiology, Department of Cardiology MC Utrecht Utrecht The Netherlands; ^6^ Division of Internal Medicine and Dermatology, Department of Nephrology and Hypertension MC Utrecht Utrecht The Netherlands; ^7^ Department of Vascular Surgery Cardiovascular Institute, Erasmus University Medical Center Rotterdam The Netherlands; ^8^ Department of Radiotherapy Erasmus University Medical Center Rotterdam The Netherlands

**Keywords:** aging, cardiovascular diseases, Ercc1, vascular remodeling

## Abstract

Cardiovascular diseases are the number one cause of death globally. The most important determinant of cardiovascular health is a person's age. Aging results in structural changes and functional decline of the cardiovascular system. DNA damage is an important contributor to the aging process, and mice with a DNA repair defect caused by Ercc1 deficiency display hypertension, vascular stiffening, and loss of vasomotor control. To determine the underlying cause, we compared important hallmarks of vascular aging in aortas of both *Ercc1* ^*Δ/−*^ and age‐matched wildtype mice. Additionally, we investigated vascular aging in 104 week old wildtype mice. *Ercc1* ^*Δ/−*^ aortas displayed arterial thickening, a loss of cells, and a discontinuous endothelial layer. Aortas of 24 week old *Ercc1* ^*Δ/−*^ mice showed phenotypical switching of vascular smooth muscle cells (VSMCs), characterized by a decrease in contractile markers and a decrease in synthetic markers at the RNA level. As well as an increase in osteogenic markers, microcalcification, and an increase in markers for damage induced stress response. This suggests that *Ercc1* ^*Δ/−*^ VSMCs undergo a stress‐induced contractile‐to‐osteogenic phenotype switch. *Ercc1* ^*Δ/−*^ aortas showed increased MMP activity, elastin fragmentation, and proteoglycan deposition, characteristic of vascular aging and indicative of age‐related extracellular matrix remodeling. The 104 week old WT mice showed loss of cells, VSMC dedifferentiation, and senescence. In conclusion, *Ercc1* ^*Δ/−*^ aortas rapidly display many characteristics of vascular aging, and thus the *Ercc1* ^*Δ/−*^ mouse is an excellent model to evaluate drugs that prevent vascular aging in a short time span at the functional, histological, and cellular level.

## Graphical abstract

*Ercc1* ^*Δ/−*^ mice are defective in DNA repair, resulting in age‐related diseases including cardiovascular aging. To determine the underlying cause, we investigated important hallmarks of vascular aging in aortas of both *Ercc1* ^*Δ/−*^ and age‐matched wildtype mice. A comparative analysis revealed arterial thickening, cell loss, and disrupted endothelial layers in *Ercc1* ^*Δ/−*^ compared to wildtype aortas. Additionally, 24‐week‐old *Ercc1* ^*Δ/−*^ mice displayed VSMC phenotypic switching, marked by altered gene expression and a stress‐induced contractile‐to‐osteogenic phenotype switch. *Ercc1* ^*Δ/−*^ aortas exhibited increased MMP activity, elastin fragmentation, and proteoglycan deposition, indicating age‐related extracellular matrix remodeling. Importantly, the *Ercc1* ^*Δ/−*^ aortas showed more overlap with human vascular aging than aged 104 week old wildtype aortas. In summary, *Ercc1* ^*Δ/−*^ mice rapidly exhibit key vascular aging characteristics, making them an ideal model to evaluate anti‐aging drugs at a functional, histological and cellular level.

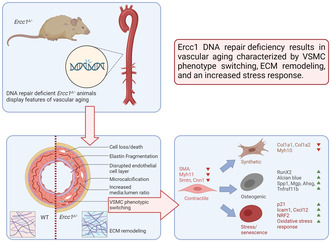

**Abbreviations**
AB: alcian blue
CVD: cardiovascular disease
DEGs: differentially expressed genes
DSB: double strand break
ECM: extra cellular matrix
HE: hematoxylin‐eosin
ICL: interstrand cross‐link
MMP: matrix metalloprotease
MYH11: myosin heavy chain 11
NER: nucleotide excision repair
NGS: normal goat serum
PDGFRβ: platelet‐derived growth factor receptor β
RF: resorcinol‐fuchsine
RUNX2: runt‐related transcription factor 2
SASP: senescence‐associated secretory phenotype
SEM: standard error of the mean
SMA: smooth muscle actin
VIM: vimentin
VSMCs: vascular smooth muscle cells
WT: wildtype

## 1 INTRODUCTION

Cardiovascular disease (CVD) is the world's leading cause of premature death, and a growing health problem. In 2016, 18 million deaths were attributed to CVD globally, which amounted to an increase of 14.5% since 2006 (Benjamin et al., 2019). A person's age is the most important determinant for the development of CVD (North & Sinclair, 2012). As the human population continues to grow older, the importance of aging as an independent risk factor for CVD, i.e., the effect of biological aging instead of the impact of cardiovascular risk factors during chronological aging, increases. Both the increasing aging population and the high mortality of CVD highlight the need to obtain more knowledge about the effects of aging on cardiovascular function. Whereas atherosclerosis mainly hinges on dyslipidemia and chronological aging as a risk factor, non‐obstructive arterial disease is observed in many animal species in association with biological aging, independently from risk factors. In case of non‐obstructive disease, high blood pressure, vascular stiffening and loss of vasomotor function are main characteristics, and are associated with increased risk for CVD (Kitta et al., 2009). Mechanistically this is driven by pathological alterations such as extracellular matrix (ECM) remodeling, dedifferentiation of vascular smooth muscle cells (VSMCs), and impaired endothelial function (Ungvari et al., 2018). Human vascular aging is characterized by structural changes such as increased media: lumen ratio, loss of VSMCs, senescence, cellular remodeling, ECM remodeling, and collagen and calcium deposition (James et al., 1995; Thijssen et al., 2016).

An important factor that enhances cellular aging is the time‐dependent accumulation of DNA damage. This accumulation is a consequence of exposure to numerous damaging agents, both exogenous, such as radiation, and endogenous, such as byproducts from metabolic activity (Durik et al., 2012; Maynard et al., 2015). Multiple DNA repair pathways exist to ensure genomic maintenance, thereby preventing accumulation of DNA damage. ERCC1 is an important protein involved in DNA repair. It forms a heterodimeric complex with a structure‐specific endonuclease (XPF) and functions in Nucleotide Excision Repair (NER), which repairs damage of cyclobutane pyrimidine dimers caused by UV irradiation (Faridounnia et al., 2018). ERCC1‐XPF cooperates with XPG in the NER pathway to excise a 25–30 nucleotide single‐stranded fragment containing the DNA damage (Faridounnia et al., 2018). Multiple studies indicate that ERCC1‐XPF also functions in other DNA repair pathways, such as double‐strand break (DSB) repair and interstrand cross‐link (ICL) repair (Faridounnia et al., 2018). As the ERCC1‐XPF complex is very important in maintaining genomic stability, deficiencies in ERCC1‐XPF result in aging phenotypes. This is demonstrated by several human progeroid syndromes, such as Cockayne syndrome and XPF‐ERCC1 syndrome (Faridounnia et al., 2018; Kashiyama et al., 2013). Likewise, mouse models carrying mutations in this complex display severe premature aging. Mice in which *Ercc1* is completely knocked out display multiple age‐related pathologies and have an extremely short life‐span of only a few weeks (Weeda et al., 1997). Besides the full knockout model, a truncated *Ercc1* mutant was generated in which the last 7 amino acids of the protein are removed. These *Ercc1* ^*Δ/−*^ mice live approximately 6 months and progressively develop multiple age‐related phenotypes that are commonly observed in elderly humans (Weeda et al., 1997). For instance, these mice suffer from cardiovascular problems such as hypertension, vascular stiffness and cardiomyopathy (Durik et al., 2012; Wilbert P. Vermeij, Hoeijmakers, & Pothof, 2016). DNA repair deficiency in the heart results in cardiac remodeling and dysfunction (de Boer et al., 2023).

Previous research in the aorta showed an increase in senescence‐associated β‐galactosidase in *Ercc1* ^*Δ/−*^ aortas (Durik et al., 2012). At the mRNA level an increase in the expression of *Cdkn2a* (*p16*), Cdkn1a (*p21*), *Nqo1* and immune factors such as *Il‐6* and *Tnf‐α* was observed (Ataei Ataabadi et al., 2022; Durik et al., 2012; Yousefzadeh et al., 2020). Furthermore, *Ercc1* ^*Δ/−*^ aortas have lower aortic contractility and a lowered endothelium‐dependent and endothelium‐independent vasorelaxant response to acetylcholine compared to wildtype (WT) littermates (Ataei Ataabadi et al., 2022; Golshiri et al., 2020). In addition, they display increased vascular stiffness (Durik et al., 2012). Thus, the model displays the prime non‐atherosclerotic physiological features of human aging‐related vascular dysfunction. The model was shown to be useful for testing anti‐aging interventions, such as phosphodiesterase 1 inhibition and dietary restriction (Golshiri et al., 2021; Vermeij, Dollé, et al., 2016; Wu et al., 2017). However, the cellular and histological remodeling mechanisms underlying these functional changes have not yet been elucidated. Similarly, the exact consequences of a DNA repair defect for the vascular system remain unclear, and more knowledge on the causative role of genomic instability in the process of vascular aging is necessary to understand the molecular mechanisms involved, and to find drug targets. To obtain more insight into the possible role of DNA repair in vascular aging in general and to assess the potential of the *Ercc1* ^*Δ/−*^ model in particular, we here interrogate the cellular and histological changes that potentially underlie the functional vascular problems.

## 2 MATERIALS AND METHODS

### 2.1 Generation and breeding experimental mice

For all experiments, *Ercc1* ^*Δ/−*^ mice, carrying a genetic mutation in which one of the alleles is removed, “knock‐out”, and the other allele has a C‐terminally truncation, “delta”, leading to the production of a truncated version of the Ercc1 protein (*Ercc1* ^*Δ/−*^), and their WT littermates were used. *Ercc1* ^*Δ/−*^ mice in an F1 hybrid FVB/C57BI6J background were obtained by crossing *Ercc1* ^*−/+*^ with *Ercc1* ^*Δ/+*^ mice of C57Bl6J and FVB background (and vice versa) (Maynard et al., 2015; Wilbert P. Vermeij, Dollé, et al., 2016; Weeda et al., 1997). Responsible local and national authorities gave permission to generate and use genetically modified animals. All experiments were performed in compliance with the “Animal Welfare Act” of the Dutch government and approved by an independent Animal Ethics Committee consulted by the Erasmus Medical Centre. The 104 week old mice were obtained in a C57BL6 background.

Mice were housed at the Animal Resource Center of the Erasmus Medical Centre. They received standard mouse food (CRM pellets, SDS BP Nutrition Ltd; gross energy content 18.36 kJ/g dry mass, digestible energy 13.4 kJ/g) and water. We studied *Ercc1* ^*Δ/−*^ mice and WT littermates at a young age of 6 weeks and at older ages of 22–24 weeks, indicated as 24 week old. Both male and female mice were used; sex‐differences were not compared due to small sample size. Information on all mice is given in Table S1.

### 2.2 Ex vivo fluorescent imaging

Mice were injected intravenously with the fluorescently labeled probes Annexin‐Vivo 750™, matrix metalloprotease (MMP) Sense680™ (Perkin Elmer, Waltham, MA, USA) or the PDGFRβ Cy7 NIRF (near infrared) probes (BiOrion, Groningen, The Netherlands). For PDGFRβ Cy7 two different probes were used: BOT5030, which recognizes and binds to the PDGFRβ, and the scrambled probe BOT5038. Mice were euthanized with an overdose of inhaled isoflurane. Aortas were collected and imaged with the Odyssey® CLx imaging system (LI‐COR® Biosciences, Lincoln, NE, USA). Probe intensity was measured with Image studio Lite version 5.20 (LI‐COR® Biosciences, Lincoln, NE, USA). Probe intensity was divided by the shape area, resulting in the intensity/mm^2^ per aorta. Total intensity was measured and divided by the surface size, *Ercc1* ^*Δ/−*^ aortas were normalized to the WT littermate aorta that was simultaneously scanned.

### 2.3 Tissue‐staining procedures

For histological analysis, mice were euthanized by CO_2_‐inhalation. Mice were perfused through the left ventricle with PBS and formalin and aortas were harvested. Aortas were fixed in formalin for 1 day and subsequently stored in 70% ethanol. Aortas were sectioned into three parts: the aortic arch, the thoracic aorta, and the abdominal aorta. The aortic arch was divided into 3 rings and the thoracic and abdominal aorta into 5–6 rings. Thereafter rings were embedded with 3% Bacto agar. The agar blocks were dehydrated through the histokinette processor (Microm) and embedded in paraffin, after which 4‐um slides were prepared. Before staining, the slides were deparaffinized in xylene and rehydrated by descending alcohol series finishing with 70% ethanol. Slides were stained with hematoxylin‐eosin (HE) for general pathology, Resorcinol‐Fuchsine (RF) for elastin structure, and Alcian blue (AB) for proteoglycan deposition of the ECM, using standard procedures. After staining, the slides were dehydrated by ascending alcohol series finishing with 100% ethanol, cleared with xylene and mounted with Pertex mounting medium.

For immunohistochemistry, the slides were first deparaffinized in xylene and rehydrated by descending alcohol series finishing with 70% ethanol. Slides for immunohistochemical staining were washed with wash buffer (1xPBS/0.3% Triton‐X‐100) and boiled in either EDTA buffer (100 mM Tris–HCl [pH 9.0] with 10 mM EDTA) or citrate buffer (10 mM Sodium citrate [pH 6.0], 0.05%Tween 20) (Table S2) at 600 W for 20 min for antigen exposure, after which slides were cooled on ice for 30 min. The endogenous peroxidase activity was blocked by incubating the slides with 3% H_2_O_2_ diluted in methanol. Then, the slides were washed with wash buffer again and blocked with 150 μL Blocking Buffer (5% normal goat serum in wash buffer) for 30 min. Slides were incubated with 150 μL of the first antibody overnight at 4°C. Used antibody conditions are indicated in Table S2.

The next day, slides were washed with wash buffer and incubated with biotinylated secondary antibody (1:200) at room temperature (RT) for 30 min. After incubation, the slides were washed again and incubated with avidin‐biotinylated complex (Vectastain Universal Elite ABC kit Vector Laboratories). After a final washing step, DAB chromogen (DAKO Liquid Dab substrate‐chromogen system) was added to each slide. The time of coloring was monitored, and slides were washed with water as soon as the section developed. Optionally, the slides were counterstained with eosin (Runt‐related transcription factor 2 (RUNX2), P21, P16, Cleaved caspase 3 and CD31). Slides were dehydrated and cleared as described above, and mounted with Pertex mounting medium.

To determine the cell number, slides were deparaffinized, rehydrated and washed with wash buffer (PBS/0.05%Tween). Slides were boiled in Citrate buffer (10 mM Sodium citrate [pH 6.0], 0.05%Tween 20) at 600 W for 20 min for antigen exposure, after which slides were cooled on ice for 30 min. Then, the slides were washed and blocked with PBS+ (PBS/5%BSA/1.5%Glycine) for 1 h at RT. After blocking, slides were incubated with 150 μL of primary antibody in PBS+ overnight at 4°C (Table S2). Slides were washed and incubated with 150 μL of secondary antibody solution (1:1000 goat anti mouse Alexa488) for 1 h at RT. Slides were washed and coverslipped with Dapi‐Vectashield.

### 2.4 Staining quantification and analysis

For quantification procedures and analysis of all stainings, qualitatively suitable sections were selected on forehand for each mouse. Sections with vague or dirty areas were excluded from quantification, and folds were removed from JPEGs to prevent false‐positive outcomes in automatic staining quantification with Fiji (ImageJ). This resulted in the analysis of 2–3 consecutive sections for the aortic arch and 3–5 consecutive sections for the abdominal and thoracic aorta per mouse, thereby covering the entire aorta.

The surface size of lumen and media was determined in HE stained slides with NDP Viewer 2. An outline of the entire section surface and of the lumen was made manually using the Freehand Region option, after which the media surface and media: lumen ratio were calculated.

In RF‐stained slides, elastin fragmentation was quantified manually. Different types of elastin fragmentation were defined before counting; elastin breaks, ending elastin fibers, and undefined disarray (Figure S1a) All fragmentation types were added together for minimal two areas (zoom 40x) of qualitatively suitable aortic sections to determine the level of fragmentation. This number was corrected for the size of the quantified area by dividing the total fragmentation count by the surface of the measured area.

Suitable aorta sections were selected for the quantification of IF staining. The number of cells and smooth muscle actin (SMA) intensity were measured by using a self‐designed macro in Fiji. In short, the aorta was selected using the SMA signal. Auto threshold (default dark) was used to generate a mask of the aorta (SMA‐mask). To measure the number of cells we generated a mask using the Dapi signal. Individual cells were selected using the adjustable watershed and select on size tools. Cells were only counted when located in the SMA‐mask. Cell number was corrected for surface size (SMA‐mask). Quantification of DAB for SMA, Myosin heavy chain 11 (MYH11) and Vimentin (VIM) and AB was performed on JPEGs made from qualitatively suitable sections using two self‐designed macro's in Fiji (ImageJ). In short, JPEGs were prepared by removing the adventitia and background to prevent interference with quantification. The macro for AB quantification was designed to remove Eosin counterstaining. Both macros first produced an overlay mask for measurement of relative stain intensity according to the total section surface. Then, the percentage of stained pixels was determined using a threshold of 173 to distinguish the stained pixels from background. The mean percentage of the DAB and Alcian Blue positive surface was determined for the aortic arch, the abdominal aorta, and the thoracic aorta. Data was visualized as the percentage positive for the protein. MYH11 signals below detection limit were given a value of 0%.

RUNX2, p21, p16 and cleaved caspase 3 stainings were quantified by counting positive (dark brown) cells in qualitatively suitable sections from the aortic arch, abdominal and thoracic aorta of each mouse. Counting was conducted by two independent researchers and corrected for the size of the counted area, after which the average count of each region of each mouse was calculated.

### 2.5 RNA sequencing

*Ercc1* ^*Δ/−*^ mice and their wildtype littermates were sacrificed at an age of 6 weeks or 24 weeks. Table S1 indicates the number of samples per group. Mice were perfused through the left ventricle with phosphate buffered saline (PBS) and the aortic arch was isolated. Aortic tissue was homogenized using the TissueLyser 24 (Shanghai Jing Xin). Total RNA and microRNA was isolated from the aortic arch using the miRNeasy Mini Kit (Qiagen). Library preparation and mRNA sequencing was performed at GenomeScan B.V. (Genomescan B.V., Leiden, The Netherlands). Libraries were paired‐end sequenced in an Illumina NovaSeq 6000 platform at a sequencing depth of 20 million reads and 150 bp read length and included Unique Molecular Identifiers (UMIs).

### 2.6 Alignment and QC

We used UMI‐Tools v1.1.2 (Smith et al., 2017) to move the UMI barcodes from separate files to the read pairs headers. We then pre‐processed the raw fastq files with fastp v0.23.2 (Chen et al., 2018). We used STAR v2.7.10a (Dobin et al., 2013) to align the samples against the mouse reference mm39 with ENSEMBL v107 annotation. After alignment, we used UMI‐Tools again to deduplicate reads originating from the same molecule and minimize the gene‐length biases derived from PCR‐amplification.

Quality Control was assessed by fastQC v0.11.9 (https://github.com/s‐andrews/FastQC/) and RSeQC v5.0.1 (Wang et al., 2016; Wang et al., 2012). MultiQC v1.15.dev0 (Ewels et al., 2016) was used to summarize the QC of these two programs and the different steps of the data processing. Read counts overlapping the entire gene body were generated using htseq‐count v2.0.2 (Putri et al., 2022).

### 2.7 Differential gene expression analysis

The resulting gene‐count matrices were analyzed with R v4.1.2 (R Core Team (2021). R: A language and environment for statistical computing. R Foundation for Statistical Computing, Vienna, Austria. URL https://www.R‐project.org/.). One sample (003, WT, female, 24 weeks, Ad Libitum) had too low read counts and diverging expression profiles, thus, it was removed from subsequent analyzes.

We used DESeq2 v1.34.0 (Love et al., 2014) to analyze differential gene expression between genotypes and conditions with an adjusted *p*‐value threshold of 0.05. The differential expression model used the sample's sex as a covariate following a design formula “~group + sex” to minimize the effect of the sample's sex on the results and focus the analysis on the actual differences groups.

### 2.8 Gene set enrichment analysis

We used the R package fGSEA v1.20.0 (https://www.biorxiv.org/content/10.1101/060012v3) to perform Gene Set Enrichment Analysis on the results of the Differential Gene Expression analysis. Each comparison between groups and conditions was analyzed separately using the log2 Fold Change of all the detected genes. Genes not present in the data were excluded from the background of the analysis.

### 2.9 IPA analysis

Normalized expression values were uploaded into Ingenuity Pathway Analysis (IPA© software version 101138820, QIAGEN, Redwood City, California, USA). An IPA core analysis was performed on significantly differentially expressed genes with a *p*‐value ≤0.05. Upstream regulators were investigated from this analysis.

### 2.10 Statistics

A one‐way ANOVA was used to compare mice with different ages and genotypes. In case of comparing 2 groups, an unpaired *t*‐test was used. To assess independent regional differences, a one‐way ANOVA was used in case of three regions. Statistical tests were conducted using Graphpad Prism 9.3.1. All results are expressed as mean ± standard error of the mean (SEM).

## 3 RESULTS

### 3.1 *Ercc1* ^*Δ*^ ^*/−*^ aorta undergoes structural changes

**FIGURE 1:**
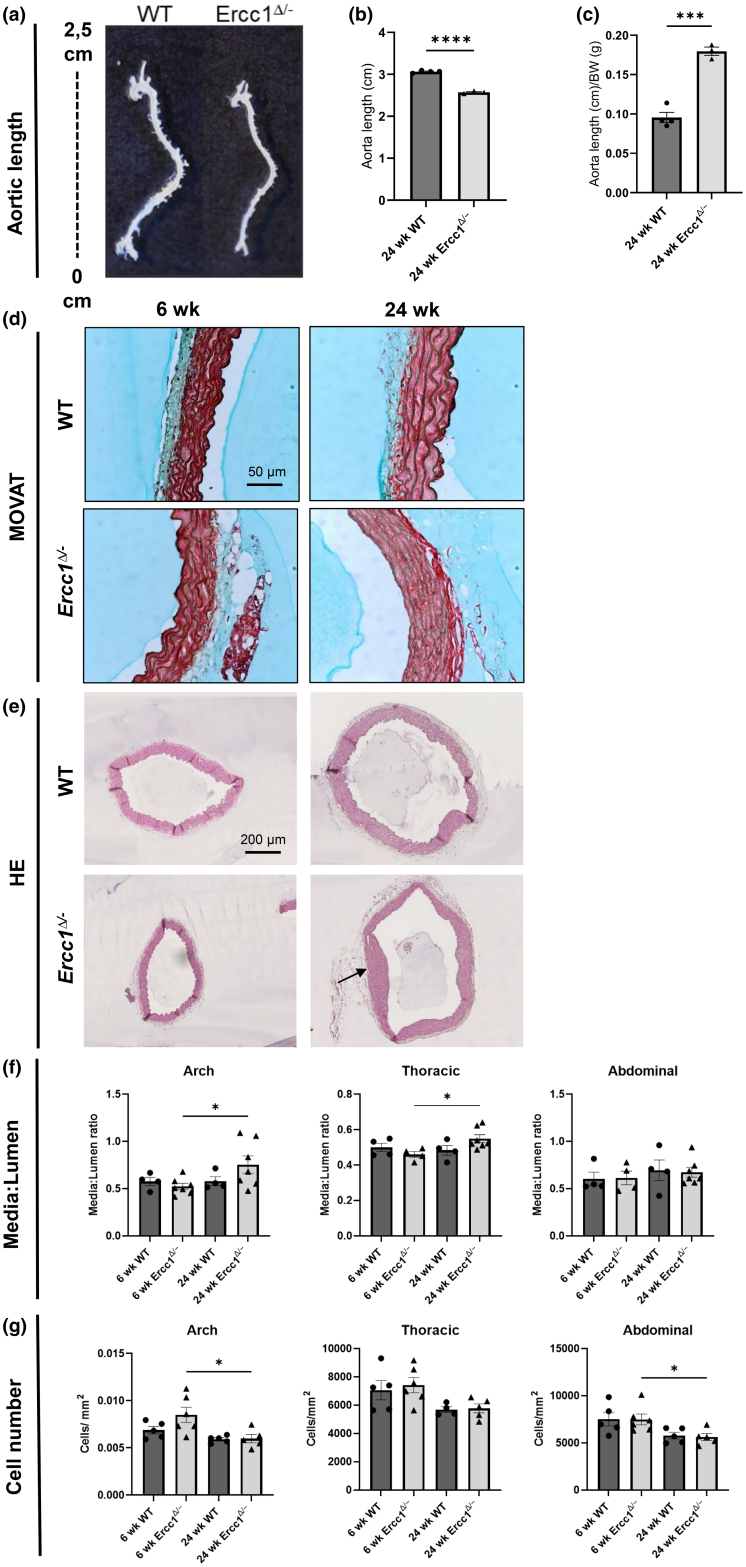
Age‐related structural changes in the *Ercc1* ^*Δ/−*^ aorta. (a–c) Quantification of the aortic length the *Ercc1* ^*Δ/−*^ mice. (a) Pictures of wildtype and *Ercc1* ^*Δ/−*^ aortas illustrating the difference in length. (b) The aortic length in *Ercc1* ^*Δ/−*^ mice is significantly shorter than that of wildtype littermates. (c) Aortic length corrected for body weight showed a significantly higher ratio in the *Ercc1* ^*Δ/−*^ mice. Scale bar = 50 μm.(d) Illustrative images of H&E stained wildtype and *Ercc1* ^*Δ/−*^ aortas. Scale bar = 200 μm. (e) media:lumen ratio of the aortic arch, thoracic aorta and abdominal aorta of Ercc1^Δ/−^ and wildtype mice. (f) The amount of cells was calculated for each aortic ring and corrected for surface area, indicating a significant decrease in cells in the 24 week old versus 6 week old *Ercc1* ^*Δ/−*^ aortas. (g) Sample sizes: MOVAT *n=3‐5*, aortic length *n* = 4, HE *n* = 4–7. Results are represented as mean ± SEM, *p < 0.05, ***p < 0.001, ****p < 0.0001.

Reduced compliance has been observed previously (Durik et al., 2012) for *Ercc1* ^*Δ/−*^ aortas, and structural remodeling could contribute to this functional deficit. To examine the effect of DNA repair deficiency on vascular aging, we first examined aortic structure. *Ercc1* ^*Δ/−*^ aortic length was 0.5 cm shorter than that of WT littermate aortas (Figure 1a,b). However, since *Ercc1* ^*Δ/−*^ mice are approximately two times smaller than WT mice (Dollé et al., 2011) aortic length of *Ercc1* ^*Δ/−*^ mice is actually longer when corrected for body weight (Figure 1a–c).

To gain more information about histological changes of the *Ercc1* ^*Δ/−*^ aortas, we performed a MOVAT staining (Figure 1d). This staining simultaneously reports on collagen, elastin, muscle, mucin and fibrin in tissue sections. The most striking findings in the 24 week old *Ercc1* ^*Δ/−*^ compared to WT littermate aortas were indications of microcalcification, disorganized and fragmented elastin fibers, increased mucin, and an increase of fibrin at the border of media and adventitia.

HE staining (Figure 1e) was used to determine the lumen surface, media surface, and media:lumen ratio in the aortic arch, the thoracic aorta, and the abdominal aorta. Absolute dimensions are presented in Figure S1b,c. Since *Ercc1* ^*Δ/−*^ mice are very small, these dimensions are in general smaller than of WT. Therefore, only media:lumen ratio can be compared between *Ercc1* ^*Δ/−*^ and WT mice. Indeed, as demonstrated in WT aortas media:lumen ratio is constant, being similar in all regions and between the 6 week old and 24 week old WT groups (Figure 1f, Figure S1b,c). 24 week old *Ercc1* ^*Δ/−*^ mice showed a significantly elevated media:lumen ratio in the aortic arch and the thoracic aorta compared to 6 week old *Ercc1* ^*Δ/−*^ mice (Figure 1f). A patchy, local thickening was occasionally observed in 24 week old *Ercc1* ^*Δ/−*^ aortas (Figure 1e arrow). The number of cells present in the aortic sections was measured in Fiji and divided by the surface size (Figure S1d). In both WT and *Ercc1* ^*Δ/−*^ aortas the cell number tended to decrease with aging (Figure 1g), and this was statistically significant in the aortic arch and abdominal aorta of the *Ercc1* ^*Δ/−*^ mice.

### 3.2 *Ercc1* ^*Δ*^ ^*/−*^ aortas display increased apoptosis

**FIGURE 2:**
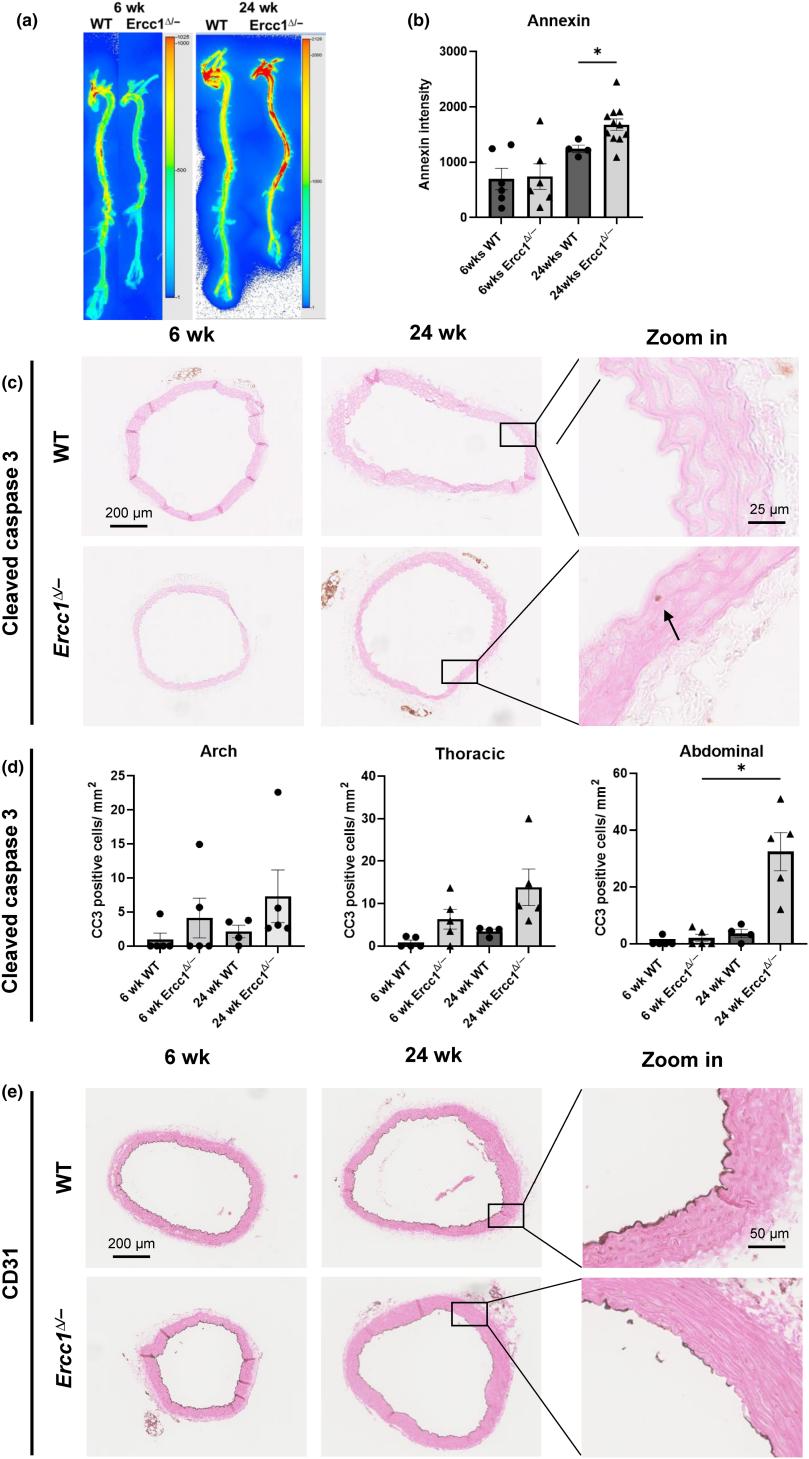
*Ercc1* ^*Δ/−*^ aortas show increased apoptosis. (a) Representative ex vivo image, of a wildtype and *Ercc1* ^*Δ/−*^ aorta injected with the Annexin‐Vivo750™. (b) Quantification of the Annexin‐Vivo750™ probe showed a significantly increased signal in *Ercc1* ^*Δ/−*^ aortas. Representative images of aortic arch of cleaved caspase 3 (c), indicating a significant increase in apoptotic cells in the abdominal aorta of the 24 week old *Ercc1* ^*Δ/−*^ (d). (e) CD31 staining showed an interrupted endothelial layer in the 24 week old *Ercc1* ^*Δ/−*^. Sample sizes: Annexin imaging *n* = 3, Cleaved caspase 3 *n* = 4–5, CD31 *n* = 3. Results are represented as mean ± SEM, *p < 0.05, ****p < 0.001. Scale bar = 25–200 μm.

To investigate whether the observed age‐related decrease in vascular cells in *Ercc1* ^*Δ/−*^ aortas is caused by apoptosis, mice were injected with the Annexin‐Vivo 750™ probe. The Annexin‐Vivo 750™ intensity showed no differences between the WT and *Ercc1* ^*Δ/−*^ aortas at 6 weeks. At 24 weeks the Annexin‐Vivo 750™ intensity was significantly increased in *Ercc1* ^*Δ/−*^ aortas compared to age‐matched WT littermates, confirming increased apoptosis in the *Ercc1* ^*Δ/−*^ aortas at 24 weeks of age (Figure 2a,b). To confirm whether indeed *Ercc1* ^*Δ/−*^ aortas show increased apoptotic cells, aortic sections were stained for cleaved caspase 3 (Figure 2c). We observed an increase in cleaved caspase 3‐positive cells in all regions of the 24 week old *Ercc1* ^*Δ/−*^ aortas (Figure 2d). This was statistically significant in the abdominal aorta of 24 week old *Ercc1* ^*Δ/−*^ compared to the 6 week old *Ercc1* ^*Δ/−*^ mice. No differences were observed between the 6 week old *Ercc1* ^*Δ/−*^ and age‐matched WT aortas. Interestingly, about half of the of cleaved caspase 3 positive cells in the aortic arch of the 24 week old *Ercc1* ^*Δ/−*^ mice were present in the endothelial layer. We therefore used CD31 staining to visualize endothelial cells. This staining showed a discontinuous endothelial layer in 24 week old *Ercc1* ^*Δ/−*^ mice (Figure 2e). Overall, these data show an age‐related increase in apoptosis in 24 week old *Ercc1* ^*Δ/−*^ aortas.

### 3.3 Ercc1^Δ^ ^/−^ VSMCs dedifferentiate from a contractile toward an osteogenic phenotype in the aortic arch

**FIGURE 3:**
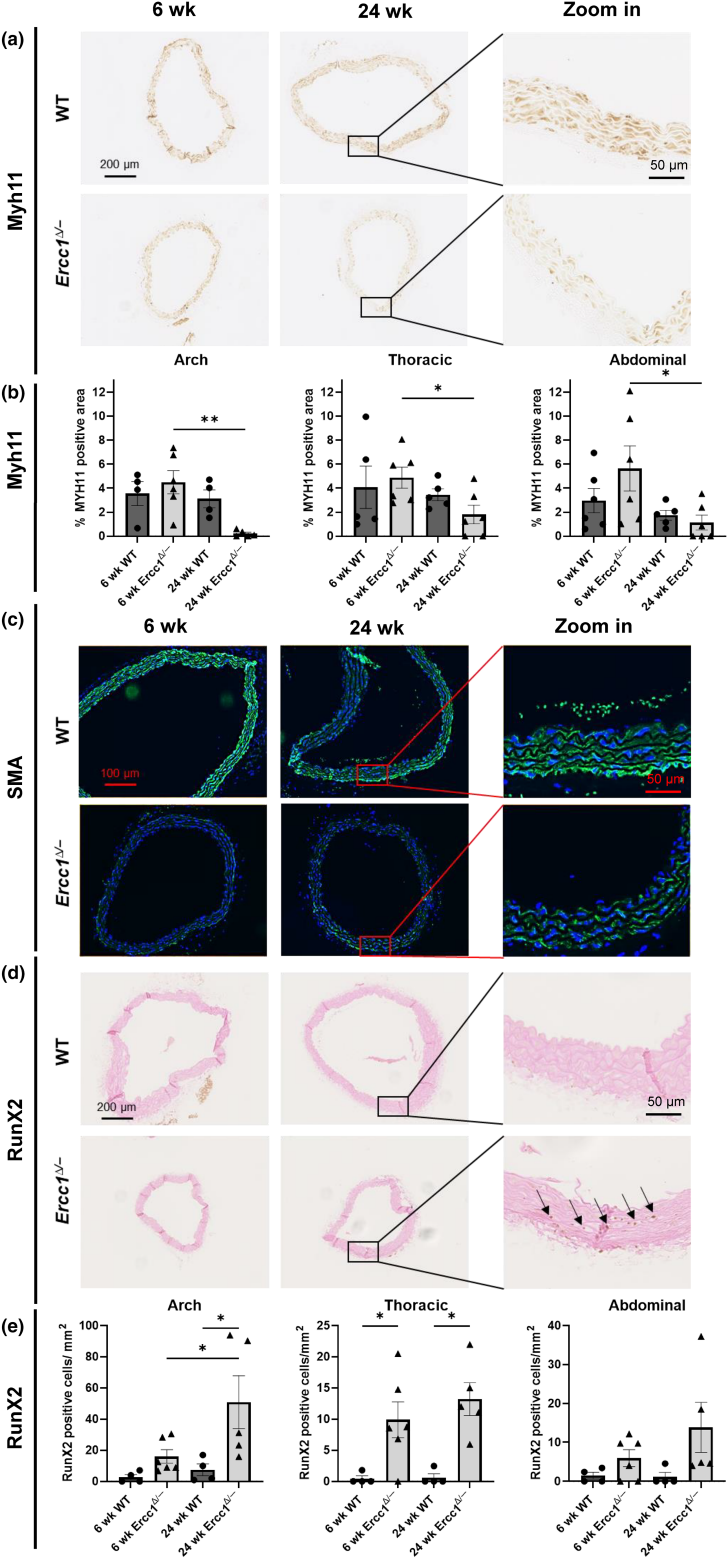
Phenotype switching of the *Ercc1* ^*Δ/−*^ VSMCs. (a) Representative images of the *Ercc1* ^*Δ/−*^ and wildtype aortic arch for MYH11. (b) The 24 week old *Ercc1* ^*Δ/−*^ aorta show a significant decrease in MYH11 staining compared 6 week old mice. (c) SMA showed a decrease in the *Ercc1* ^*Δ/−*^ aortas (both ages) compared to their age‐matched wildtype littermates. (d) Representative images of the *Ercc1* ^*Δ/−*^ and wildtype aortic arch for RUNX2 staining. (e) Quantification of RUNX2 showed a significant increase in the 24 week old *Ercc1* ^*Δ/−*^ aortic arch, and at both 6 week old and 24 week old age in the thoracic aorta compared to wildtype littermates. Sample sizes: MYH11 *n* = 5–6, SMA *n* = 5, RUNX2 *n* = 4–6. Results are represented as mean ± SEM, *p < 0.05, **p < 0.01. Scale bar = 50–200 μm.

A reduction in vasomotor dysfunction could be explained by dedifferentiation of VSMCs (Touyz et al., 2018). To explore age‐related VSMC dedifferentiation, aorta tissue sections were stained for MYH11, SMA (for contractile VSMCs), VIM (for synthetic VSMCs) and RUNX2 (for an osteogenic phenotype). The MYH11 signal decreased at all sites in 24 week old versus 6 week old *Ercc1* ^*Δ/−*^, and this reached significance in the aortic arch and abdominal aorta (Figure 3a,b). Such decreases did not occur in the age‐matched WT mice.

The IF staining for SMA did not show a continuous staining for *Ercc1* ^*Δ/−*^ aortas. Quantification of the SMA signal was not possible due to the fluorescent signal of the elastin layers. However, representative images of the aortas show a decrease of SMA signal in the 6 week old and 24 week old *Ercc1* ^*Δ/−*^ aorta compared to their age‐matched WT littermates (Figure 3c).

Loss of contractile markers was further confirmed by RNAseq data, showing significant downregulation of *Acta2*, the gene encoding for SMA, in both the 6 and 24 week old *Ercc1* ^*Δ/−*^ aortic arches compared to WT (Figure S3a,b and Figure S4a). *Myh11* was significantly downregulated in the 24 week old *Ercc1* ^*Δ/−*^ arch compared to their age‐matched WT littermate (Figure S3a, and Figure S4a).

Next, vimentin staining showed local areas with less (or no) staining in the aortic arch of 24 week old *Ercc1* ^*Δ/−*^ aortas (Figure S2a). However, when quantifying the total vimentin signal, no significant differences were observed (Figure S2b). The gene encoding for vimentin (*Vim*) was not significantly changed between 24 week old WT and *Ercc1* ^*Δ/−*^ aortas (Figure S4b). However, RNAseq data did reveal a decrease in the synthetic markers; *Col1a1*, *Col1a2* and *Myh10* in the 24 week old *Ercc1* ^*Δ/−*^ arches compared to WT littermates and compared to 6 week old *Ercc1* ^*Δ/−*^ arches (Figure S4b). Finally, WT aortas contained only a few RUNX2 positive cells. RUNX2 positivity increased greatly in *Ercc1* ^*Δ/−*^ aortas, although significance was reached only in the thoracic aorta (Figure 3d,e). The highest amount of RUNX2 positive cells were observed in the aortic arch. RUNX2 positive cells were often observed to be localized near each other. Interestingly, RNAseq data revealed that normalized counts of *Runx2* were low (<20) and no significant difference in *Runx2* expression was observed between the *Ercc1* ^*Δ/−*^ and WT aortic arch (data not shown). Nonetheless, RNAseq data suggested vascular calcification, showing a significant upregulation of *Spp1*, *Mgp*, *Ahsg*, *Tnfrsf11a* and *Tnfrsf11b*, genes encoding for proteins involved in calcification (Figure S3 and Figure S4c) (Escobar Guzman et al., 2020). Taken together, these data suggest that the decrease of contractile markers in the 24 week old *Ercc1* ^*Δ/−*^ aortas is due to a VSMC phenotypical switch toward a more osteogenic state, which is further substantiated by the increased microcalcification as observed in the MOVAT staining.

### 3.4 *Ercc1* ^*Δ*^ ^*/−*^ aortas show an increased damage induced stress response

**FIGURE 4:**
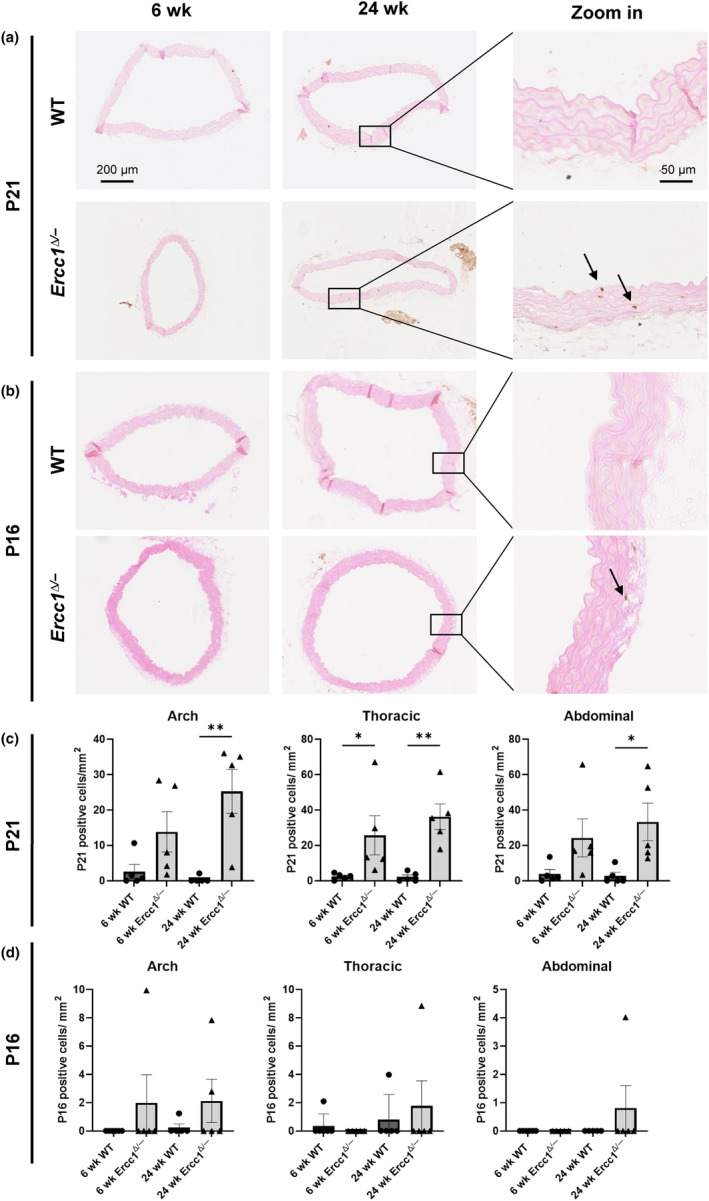
*Ercc1* ^*Δ/−*^ VSMCs show increased senescence. Representative images of the *Ercc1* ^*Δ/−*^ and wildtype aortic arch for p21 (a), and p16 (b) staining. (c) Quantification of p21 staining shows a significant increase in *Ercc1* ^*Δ/−*^ aortas, compared to wildtype littermates. (d) Quantification of the p16 staining showed a non‐significant increase in the 24 week old *Ercc1* ^*Δ/−*^ aortas, although no significance was reached due to small amount of p16 positive cells which made quantification unreliable. Sample sizes: p21 *n* = 5, p16 *n* = 5–6. Results are represented as mean ± SEM, *p < 0.05, **p < 0.01. Scale bar = 50–200 μm.

In order to examine cellular stress and senescence in both endothelial cells and VSMCs, aortic sections were stained for p21 and p16 (Figure 4a,b). P21 is a marker for DNA damage response, and is associated with cellular senescence and stress. In *Ercc1* ^*Δ/−*^ aortas, p21 positive cells were increased in both 6 week old and 24 week old mice versus age‐matched WT littermates. This was significant in the thoracic aorta for the 6 week old mouse group, and at all 3 regions in the 24 week old mouse group (Figure 4c). We only observed a small amount of p16 positive cells, which made quantification unreliable (Figure 4d). RNAseq data showed a significant upregulation of *Cdkn1a*, the gene encoding for p21, in the *Ercc1* ^*Δ/−*^ aortic arch at both ages compared to their age‐matched WT littermates (Figure S3 and Figure S4d). RNAseq data of *Cdkn2a* (p16) revealed a low number of normalized counts (<10) for all samples (Figure S4d). To further investigate senescence, we explored the senescence‐associated secretory phenotype (SASP). Only 11.4% of the total SASP genes were significantly upregulated (data not shown). Among these upregulated genes are *Icam1*, *Mmp3* and *Cxcl12* (Figure S4e,f). As *Cdkn1a* (p21) is also involved in the cellular response to stress, such as oxidative stress, we next investigated the oxidative stress response in our RNAseq data. GSEA analysis of 24 week old WT vs *Ercc1* ^*Δ/−*^ aortas showed significant enrichment of the “oxidative stress and redox pathway” and “oxidative damage response” (Figure S5a). Both pathways contain mostly upregulated differentially expressed genes (DEGs), suggesting activation of these pathways. Additionally, upstream regulator analysis revealed predicted activation of NFE2L2 (NRF2), a transcription factor regulating the expression of antioxidant proteins, and HIF1A, a transcription factor regulating the response to reactive oxygen species (Figure S5b). Taken together, our data suggests that *Ercc1* ^*Δ/−*^ aortas show signs of an increased damage induced stress response.

### 3.5 ECM remodeling is increased in *Ercc1* ^*Δ*^ ^*/−*^ mice and is most prominent in the aortic arch

**FIGURE 5:**
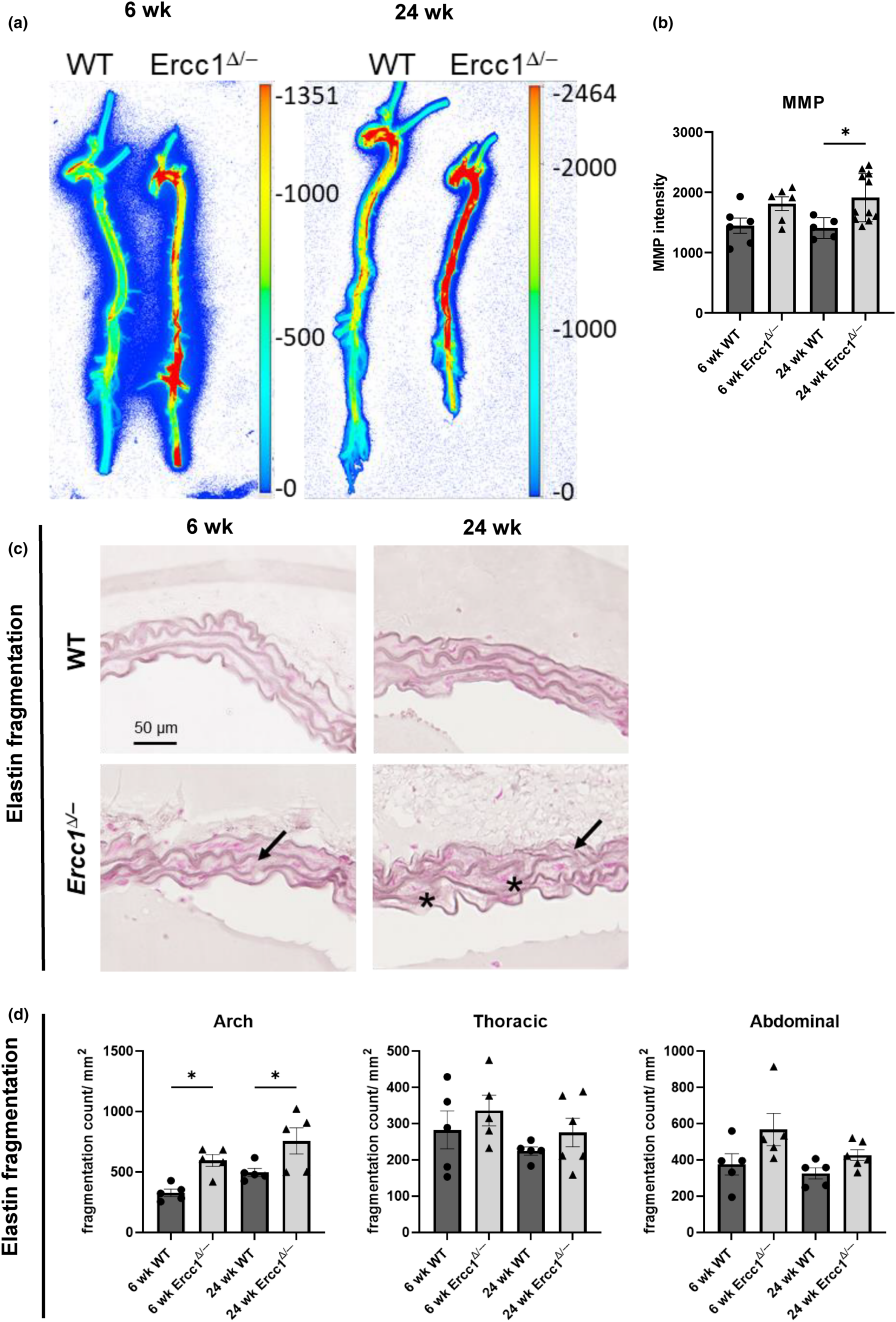
Increased MMP activity and elastin fragmentation in *Ercc1* ^*Δ/−*^ aortas. (a) Representative ex vivo image, of a wildtype and *Ercc1* ^*Δ/−*^ aorta injected with MMPsense680™. (b) Quantification of the fluorescent signal indicative of MMP activity showed a significant increase in the 6 week old and 24 week old *Ercc1* ^*Δ/−*^ aortas compared to wildtype littermates. (c) Representative images of the *Ercc1* ^*Δ/−*^ and wildtype aortic arch of the resorcin fuchsin (RF) staining used to visualize elastin. (d) Quantifications showing a significant increase in elastin fragmentation in the 6 week old and 24 week old *Ercc1* ^*Δ/−*^ aortic arch compared to wildtype littermates. Sample sizes: MMP imaging *n* = 3, RF *n* = 5. Results are represented as mean ± SEM, *p < 0.05, **p < 0.01. Scale bar = 50 μm.

Another important characteristic of vascular aging is ECM remodeling, which can affect the compliance of the vessel and the vasomotor response. ECM remodeling is partially driven by dedifferentiated VSMCs, which secrete more ECM proteins. Important enzymes in ECM remodeling are MMPs. MMPs target a wide range of ECM proteins, such as elastin and proteoglycans. Indeed, molecular imaging after injection with MMPsense680™ revealed a significant increase in MMP activity in the 24 week old *Ercc1* ^*Δ/−*^ aortas compared to age‐matched WT littermates (Figure 5a,b). This increase was significant in the full length aorta, as well as the aorta and thoracic region (Figure S2c).

In agreement with the increased activity of MMP, RNAseq data shows that both *Mmp2* and *Mmp3* are significantly upregulated in the 24 week old *Ercc1* ^*Δ/−*^ aortic arch compared to WT (Figures S3a and S4e). Additionally, RNA expression levels of the ECM remodeling regulators *Timp1* and *Timp4* are significantly increased at 24 weeks in the *Ercc1* ^*Δ/−*^ aortic arch (Figures S3a and S4e).

RF staining was used to quantify MMP‐induced elastin fragmentation (Figure 5c,d and Figure S1a). Aortic elastin fragmentation tended to be increased in both 6 week old and 24 week old *Ercc1* ^*Δ/−*^ mice versus age‐matched WT littermates, and this was significant in the aortic arch of 6 week old *Ercc1* ^*Δ/−*^ mice. The elastin network in the 24 week old *Ercc1* ^*Δ/−*^ mice was less structured compared to age‐matched WT mice (Figure 5c), matching the results of the MOVAT staining. Elastin fragmentation was lowest in the thoracic aorta.

**FIGURE 6:**
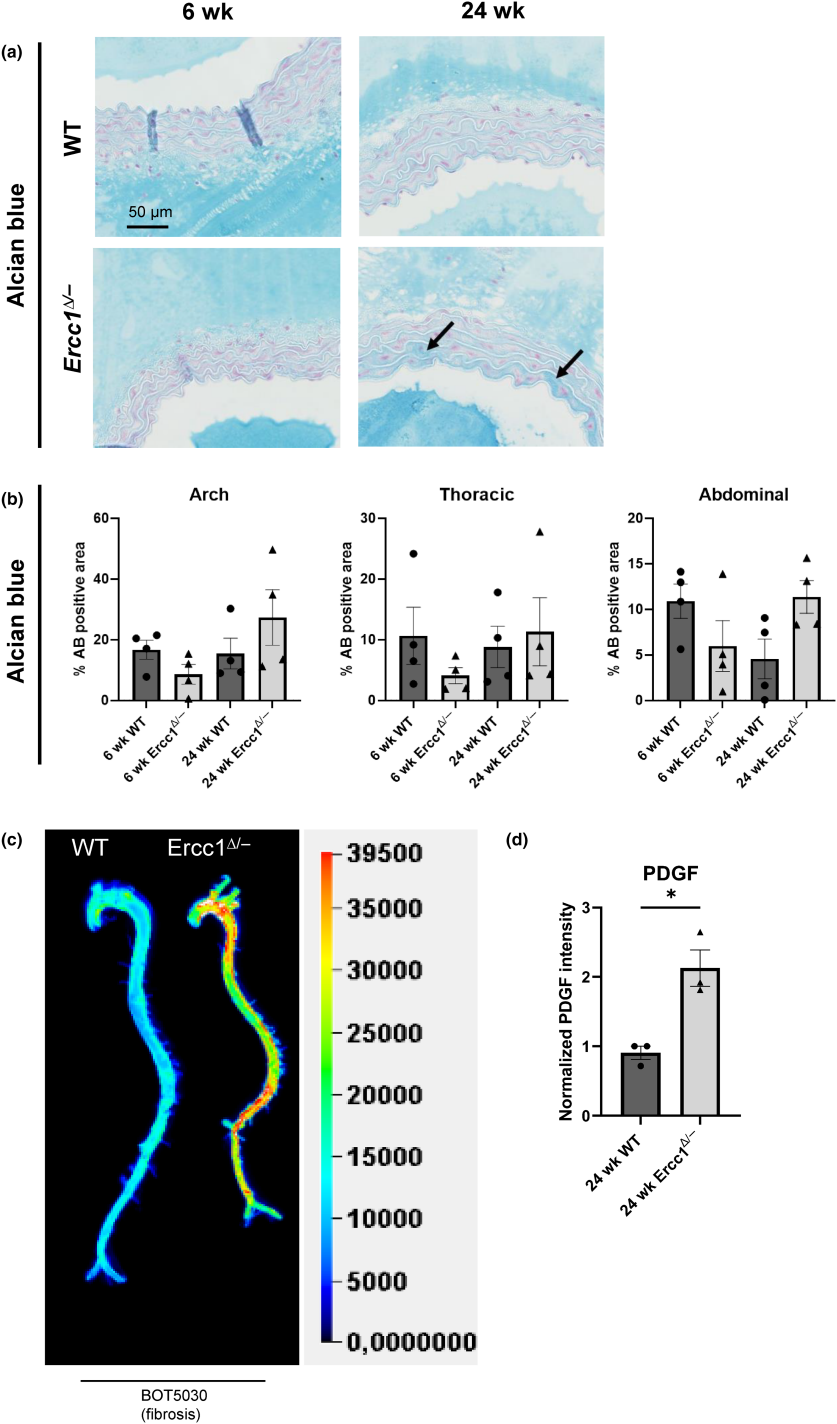
Increased alcian blue staining and PDGFRβ signal in *Ercc1* ^*Δ/−*^ aortas. (a) Representative images of the *Ercc1* ^*Δ/−*^ and wildtype aortic arch stained with alcian blue. Arrows indicate a regional increase of the staining. (b) Quantification revealed a (non‐significant) increase of alcian blue (AB) in the 24 week old *Ercc1* ^*Δ/−*^ aortas compared to the 6 week old *Ercc1* ^*Δ/−*^ aortas. (c) Representative ex vivo image, of a wildtype and *Ercc1* ^*Δ/−*^ aorta injected with the PDGFRβ probe (BOT5030). (d) Quantification showed a significant increase in PDGFRβ signal in the *Ercc1* ^*Δ/−*^ aortas when compared to wildtype littermate (d). Sample sizes: AB *n* = 4, PDGFRβ imaging *n* = 3. Results are represented as mean ± SEM, *p < 0.05. Scale bar = 50 μm.

Furthermore, the MOVAT staining indicated an increase in mucin. To further investigate this we performed an Alcian Blue staining to visualize proteoglycan deposition. The *Ercc1* ^*Δ/−*^ aortas showed an increase of proteoglycan deposition with age (Figure 6a,b). However, significance was not reached, most probably due to patchy increases in AB staining. Proteoglycan deposition was highest in the aortic arch.

Lastly, ECM remodeling and dedifferentiation of VSMCs can lead to stiffening of the vessels. This can also result in pathological conditions such as fibrosis. A precursor of fibrosis is the increase of myofibroblasts, for which an increase in the platelet‐derived growth factor receptor β (PDGFRβ) is indicative. Molecular imaging after injection of the PDGFRβ Cy7 probe revealed PDGFRβ upregulation in *Ercc1* ^*Δ/−*^ versus WT aortas (Figure 6c,d), while with the scrambled probe (BOT5038) which contains fluorescence but lacks the capacity to bind, no such increases were seen. No differences were observed in 6 week old *Ercc1* ^*Δ/−*^ versus age‐matched WT aortas (data not shown). The increased PDGFRβ signal in the 24 week old *Ercc1* ^*Δ/−*^ aortas indicates the occurrence of an initial stage of fibrosis. Additionally the observed increase of fibrin in the MOVAT staining supports fibrosis (Chambers, 2008).

In summary, *Ercc1* ^*Δ/−*^ aortas undergo increased ECM remodeling, indicated by higher MMP activity levels, elastin fragmentation, patchy increased proteoglycan deposition and an initial stage of fibrosis.

### 3.6 *Ercc1* ^*Δ*^ ^*/−*^ aortas show more overlap with human vascular aging compared to 104 week old WT mice

To further determine if the *Ercc1* ^*Δ/−*^ mice are a suitable model to study vascular aging, we compared the aortas of naturally aged 104 week old WT mice to the young 6 and 24 week old wildtype aortas, to determine which age related vascular changes reported in literature, and examined in *Ercc1* ^*Δ/−*^ mice, these old WT mice exhibit. First, we examined structural changes by measuring the media:lumen ratio. This ratio was unaffected in the 104 week aortas compared to the younger wildtypes (Figure S6a). Interestingly, 24 week old *Ercc1* ^*Δ/−*^ aortas showed a significant increase in media:lumen ratio compared to WT littermates and 6 week old *Ercc1* ^*Δ/−*^ aortas (Figure S6a and Figure 1e). The 104 week old WT aortas showed a decrease in cell number (Figure S6b) compared to younger littermates, which was similar for the 24 week old *Ercc1* ^*Δ/−*^ aortas compared to the 6 week old *Ercc1* ^*Δ/−*^ aortas (Figure 1f). The decrease in cell number was further investigated by cleaved caspase 3 staining, showing a significant increase in apoptotic cells in the 104 week old aortas compared to 6 week old WT (Figure S6c). Aortas of 24 week old *Ercc1* ^*Δ/−*^ mice also showed a slight but non‐significant increase in cleaved caspase 3 positive cells compared to younger *Ercc1* ^*Δ/−*^ mice (Figure 2d). The endothelial layer of the 104 week old WT aortas remained intact similar to younger littermates (Figure S6d), whereas the 24 week old *Ercc1* ^*Δ/−*^ aortas displayed a discontinued endothelial layer (Figure 2e). To investigate whether the aorta of 104 week old mice undergoes ECM remodeling, an RF and Alcian Blue stainings were analyzed. Interestingly 104 week old aortas did not show an increase in elastin fragmentation or proteoglycan deposition compared to younger littermates (Figure S6e,f). The 24 week old *Ercc1* ^*Δ/−*^ aortas showed a slight non‐significant increase of both elastin fragmentation and alcian blue compared to 6 week old *Ercc1* ^*Δ/−*^ aortas, indicating increased ECM remodeling (Figure 5c, Figure 6a,b).

We next examined markers of VSMC dedifferentiation in the 104 week old WT aortas. These aortas showed a decrease of contractile markers compared to younger wildtype aortas, similar to 24 week old *Ercc1* ^*Δ/−*^ aortas (Figure S7a,b, Figure 3a–c). No significant changes were observed in Vimentin in the 104 week old aortas compared to younger littermates, which is similar to the 24 week old *Ercc1* ^*Δ/−*^ aortas (Figure S7c, Figure S2). RunX2 was significantly increased in the 104 week old aortas compared to 6 week old wildtype (Figure S7d), in agreement with the 24 week old *Ercc1* ^*Δ/−*^ aorta data (Figure 3d,e). To investigate stress and senescence, p21 and p16 were analyzed. This showed increased p21 expression with high variation between samples and significantly increased p16 expression in the 104 week old aortas compared to 6 week old WT aortas (Figure S7e,f). Interestingly, a significant increase in p16 staining was not observed in the 24 week old *Ercc1* ^*Δ/−*^ aortas (Figure 4d).

Overall, our data indicate that both the 104 week old WT mice and the Ercc1^Δ/−^ mice show characteristics of vascular aging. The Ercc1^Δ/−^ aortas shows more overlap with human vascular aging, indicated by medial thickening, loss of cells, disruption of the endothelial layer, VSMC dedifferentiation, ECM remodeling and fibrosis. The 104 week old WT mice showed loss of cells, VSMC dedifferentiation and senescence, however they lack several vascular aging characteristic which are displayed in the Ercc1^Δ/−^ aortas.

## 4 DISCUSSION

This study has investigated characteristics of vascular aging in DNA repair deficient *Ercc1* ^*Δ/−*^ aortas and age‐matched WT aortas. Additionally, we investigated these characteristics in 104 week old WT mice and young WT mice. Our results indicate that *Ercc1* ^*Δ/−*^ aortas show more overlap with human vascular aging compared to aortas of naturally aged mice. It was previously shown that *Ercc1* deficient mice display accelerated cardiovascular aging, evidenced by hypertension and vascular stiffness (Durik et al., 2012). Our data now show that at the structural and cellular level this phenotype is multifactorial, involving aortic lengthening, increased apoptosis, VSMC dedifferentiation from a contractile to an osteogenic phenotype, increased stress, proteoglycan deposition, elastin fragmentation and fibrosis. MMP and PDGFRβ increase are mechanistic explanations for the extracellular matrix changes.

Aging results in structural and functional changes of the aorta. These changes include an increase of the aortic length (Hopper et al., 2021) and thickening of the arterial wall, resulting in an increased media:lumen ratio (Bia et al., 2011; Jani & Rajkumar, 2006).

The decrease in cell number in the aortic wall that is observed during aging likely involves apoptosis (Monk & George, 2015). Aging promotes programmed cell death pathways, such as apoptosis and necroptosis (Ungvari et al., 2018). It has previously been shown that VSMCs in the inner half of the aortic media in elderly humans undergo apoptosis (Sawabe, 2010). Also, in aged rats the number of apoptotic endothelial cells in coronary arteries is increased (Csiszar et al., 2004; Ungvari et al., 2018). This will result in a loss of cells with aging (López‐Otín et al., 2013). Here it is important to note that the vascular cell numbers in 6 week old WT and *Ercc1* ^*Δ/−*^ aortas were similar, suggesting that the observed cell loss in the 24 week old *Ercc1* ^*Δ/−*^ aortas is not due to a developmental defect, but rather reflects an age‐related phenotype caused by the ERCC1 mutation. Overall these data indicate an overlap between the *Ercc1* ^*Δ/−*^ mouse and human vascular aging, both showing increased apoptosis.

In concordance, by use of the Annexin‐Vivo 750™ probe, we also observed increased apoptosis in *Ercc1* ^*Δ/−*^ aortas. Moreover, cleaved caspase 3 staining appeared to be increased in the endothelial layer of the *Ercc1* ^*Δ/−*^ aortic arch. In agreement with endothelial apoptosis, CD31 staining revealed that the endothelial layer in *Ercc1* ^*Δ/−*^ aortas was discontinuous. A potential cause of endothelial apoptosis is endothelial cell stress (Bautista‐Niño et al., 2020). Taken together, *Ercc1* ^*Δ/−*^ aortas show increased apoptosis, concentrated in the endothelial cell layer, most probably leading to disruption of this layer with age.

VSMCs are described to undergo a phenotypical switch with aging (Lacolley et al., 2018; Petsophonsakul et al., 2019). Depending on their environment, this involves dedifferentiation from a contractile phenotype to a synthetic, osteogenic, senescent, foam cell‐like, macrophage cell‐like, or myofibroblast‐like phenotype (Monk & George, 2015; Ribeiro‐Silva et al., 2021; Sorokin et al., 2020). Contractile VSMCs are quiescent and express contractile proteins, such as SMA, SM22 and MYH11. Synthetic cells are characterized by proliferation and migration. Furthermore, they express lower levels of proteins involved in contraction and produce more pro‐inflammatory cytokines and elastolytic enzymes, such as MMPs. This results in, among other things, fragmentation of the elastin network (Lacolley et al., 2018; Petsophonsakul et al., 2019). Osteogenic VSMCs down‐regulate contractile markers and express bone markers like RUNX2 and osteopontin, and contribute to calcification and proteoglycan deposition (Cao et al., 2022; Monk & George, 2015). Aging affects the homeostasis of VSMCs, which can also result in senescence. These senescent VSMCs display altered secretory activities (SASP), and includes the secretion of inflammatory factors and MMPs. An upregulation of osteogenic markers might also be observed (Badi et al., 2018; Cao et al., 2022). Simultaneously, senescent VSMCs display increased expression of cytoskeleton proteins, while in synthetic VSMCs the opposite occurs.

In 24 week old *Ercc1* ^*Δ/−*^ aortas we observed a decrease of contractile markers MYH11 and SMA. There was no change in the synthetic marker vimentin, while the osteogenic marker RUNX2 and the stress marker p21 were upregulated. This implies dedifferentiation from the contractile phenotype toward the osteogenic phenotype. The finding is in direct agreement with a previous observation that senescence is associated with RUNX2 upregulation in cultured VSMC and in the *klotho* knockout mouse model of accelerated aging and vascular calcification (Replicative senescence of vascular smooth muscle cells enhances the calcification through initiating the osteoblastic transition (Nakano‐Kurimoto et al., 2009)). Thus, our results indicate parallels between these models despite them being based on very different causal mechanisms. It was previously found that the *Ercc1* ^*Δ/−*^ mouse aorta shows an increase in *Cdkn2a* (p16) and *Cdkn1a* (p21) expression as well as increased SA‐β‐gal staining, implying an increase in senescence (Durik et al., 2012; Yousefzadeh et al., 2020). Despite our observation of increased p21 expression our data did not reflect the increase of other senescence markers as distinctly, but did suggest an increased cellular stress response. Next to that, the aorta of mice selectively lacking *Ercc1* in VSMCs also displayed increased mRNA expression of the genes encoding *p16* and *p21*, combined with arterial stiffening (Ataei Ataabadi et al., 2021). We observed that the *Ercc1* ^*Δ/−*^ aortas show signs of an oxidative damage induced stress response. Interestingly, the NRF2 pathway, also known as antioxidant response, was previously shown to be increased in liver of the ERCC1^*Δ/−*^ mice and CSB/XPA mice (Niedernhofer et al., 2006; Schumacher et al., 2008; van der Pluijm et al., 2006).The observed arterial stiffening in the SMC‐KO mice likely reflects calcium deposition and proteoglycan deposition, in agreement with the osteogenic phenotype and the increased AB staining observed in the present study. Additionally, stiffening is often accompanied by fibrosis. An early signal of fibrosis is increased PDGFRβ signaling in myofibroblasts (Li et al., 2021; Omarjee et al., 2019; Shao et al., 2011). Our data showed an increase in the PDGFRβ Cy7 probe in the 24 week old *Ercc1* ^*Δ/−*^ aortas, supporting the concept that fibrosis is increased in *Ercc1* ^*Δ/−*^ aortas (Li et al., 2021; Omarjee et al., 2019; Shao et al., 2011) Taken together, aging causes contractile VSMCs to dedifferentiate to VSMCs with an osteogenic phenotype, resulting in ECM remodeling, calcification, proteoglycan deposition, and fibrosis, and thus contributing to vascular stiffening.

ECM remodeling can affect the differentiation of VSMCs and in turn dedifferentiated VSMCs secrete ECM factors. In our study, ECM remodeling is not only reflected by proteoglycan deposition, but also by increased expression of MMP and elastin fragmentation in *Ercc1* ^*Δ/−*^ aortas. The aortic arch was the most affected region. This might be explained by the fact that this area experiences the highest blood pressure. Our data shows that MMPs and elastin fragmentation are already increased in the 6 week old *Ercc1* ^*Δ/−*^ aortas, suggesting that ECM remodeling precedes VSMC dedifferentiation. Elastin‐derived peptides, generated by elastase, promote dedifferentiation of VSMCs toward the osteogenic phenotype. Which was also observed by the increase of RUNX2 in the 24 week old *Ercc1* ^*Δ/−*^ aorta. In turn, osteogenic VSMCs secrete ECM molecules (such as collagens and proteoglycans), which tended to be increased in 24 week old *Ercc1* ^*Δ/−*^ aortas (Ribeiro‐Silva et al., 2021).

A remaining question is why genomic instability causes rapid vascular aging. The following mechanisms could explain this. First, decreased efficiency of repair leads to presence of unrepaired DNA damage, which as a consequence upregulates p53 and p73 protein expression. These proteins are key factors contributing to ECM remodeling, for instance by increasing the expression of ECM genes such as FN1 (Yokoi et al., 2020). Second, one of the proteins upregulated in 24 week old *Ercc1* ^*Δ/−*^ aortas is RUNX2, which has previously been linked to genomic instability (Duer et al., 2020). DNA damage results in the upregulation of PARP signaling, which suppresses miRNA‐204 via the IL‐6/STAT3 pathway. RUNX2 is a target of miRNA‐204 and suppression of miRNA‐204 leads to an increase of RUNX2 (Wang et al., 2019). Recent research suggests a more direct role of RUNX2 in the DNA damage response. RUNX2 is involved in the nuclear matrix, which is important in transcription, apoptosis, DNA replication and DNA repair (Yang et al., 2015). The increase of RUNX2 in turn promotes osteogenic differentiation, apoptosis and calcification (Duer et al., 2020). RUNX2 has also been linked to oxidative stress, which we observed in the RNAseq data. Oxidative stress activates downstream pathways, including AKT, which contributes to RUNX2 transcriptional activity (Byon et al., 2008; Cohen‐Solal et al., 2015). Additionally, genomic instability can have harmful consequences, such as aging. To prevent this, organisms develop protective mechanisms like apoptosis and senescence (Bautista‐Niño et al., 2016). The loss of cells as well as the increase in p21 are likely to affect both ECM remodeling and VSMC dedifferentiation. In the *Ercc1* ^*Δ/−*^ aortas we observed a direct link with oxidative stress, which also affects ECM remodeling.

Human vascular aging is characterized by structural changes such as increased media:lumen ratio, loss of VSMCs, senescence, cellular remodeling, ECM remodeling, and collagen and calcium deposition (James et al., 1995; Thijssen et al., 2016). All these characteristics were observed in the *Ercc1* ^*Δ/−*^ aortas. Furthermore, physiological changes observed in human vascular aging include endothelial dysfunction, arterial stiffening and high blood pressure (Dai et al., 2015), which are also observed in the *Ercc1* ^*Δ/−*^ mice (Durik et al., 2012). A total of three patients were documented to possess an *ERCC1* mutation (XFE patients), which all died before reaching the age of 3 (Baer et al., 2020; Jaspers et al., 2007; K Kashiyama et al., 2013). There were no reports of vascular aging phenotypes associated with these cases. It is unknown whether these patients underwent vascular assessments during their early years (van der Linden et al., 2023).

Vascular aging has also been studied in other accelerated aging mouse models with genome instability. A well‐known model is the *Lmna* ^*G609/G609*^ mouse model, which showed vascular stiffness, ECM remodeling, VSMC depletion and changes to the elastin structure (del Campo et al., 2019; Osorio et al., 2011). Another well‐studied mouse model is the *BubR1* mice (Matsumoto et al., 2007), show endothelial dysfunction, decreased levels of elastin, fibrosis, thinning of both the arterial wall and the inner diameter, VSMC loss, and impaired angiogenesis. These characteristics of vascular aging were also observed in the *Ercc1* ^*Δ/−*^ aortas (van der Linden et al., 2023). However, the *Ercc1* ^*Δ/−*^ aortas showed even more characteristics of human vascular aging, including VSMC dedifferentiation and stress.

In conclusion, healthy cells normally respond to DNA damage by repairing the damage. The DNA repair deficient *Ercc1* ^*Δ/−*^ mouse lacks this capacity, and thus experiences genomic instability. As a consequence, apoptosis and DNA damage induced stress occur, among others resulting in RUNX2 upregulation. This eventually results in ECM remodeling, calcification and VSMC dedifferentiation. The *Ercc1* ^*Δ/−*^ mouse is an excellent model to study these phenomena in shortened timespan, allowing the rapid evaluation of potential novel protective drugs that prevent the development of this vascular aging phenotype.

## 5 LIMITATION OF THIS STUDY

Due to the limited group of mice used, we were not able to investigate sex‐differences. Another limitation is that the 104 week old WT and the *Ercc1* ^*Δ/−*^ mice have a different genetic background and could therefore not be directly compared to each other.

## AUTHOR CONTRIBUTIONS

J.L., I.P. conceptualized and designed the project. J.L. Performed the experiment, collected data, analyzed data, and prepared figures. S.S. and J.M.H.G. analyzed RNA sequencing data. I.B. assisted with experiments and data analysis. R.M.C.B. managed mouse work. Y.R., J.N.V., B.S.T., performed molecular imaging experiments. H.S. designed and provided the PDGFRβ Cy7 probes, J.L., I.P., S.S., C.C., A.J.M.R., A.H.J.D., J.E. wrote and edited the manuscript.

## FUNDING INFORMATION

This work was supported by the Erasmus MC HDMA grant LSHM 21075. Part of the material costs were funded by TKI‐LSH grant EMCLSH 19013 to A.J.M.R.

JMHG acknowledges financial support from the National Institute of Health (NIH)/National Institute of Aging (NIA) (P01 AG017242) and ZonMW Memorabel project ID 733050810.

## CONFLICT OF INTEREST STATEMENT

There were no conflicts of interest.

## Supporting information


Figures S1–S7.

Tables S1–S2.

## Data Availability

The data that support the findings of this study are available from the corresponding author, upon reasonable request.
