# Supplementary material for: Ercc1 DNA repair deficiency results in vascular aging characterized by VSMC phenotype switching, ECM remodeling, and an increased stress response
Source: Aging Cell. 2024 Mar 7;23(5):e14126. doi: 10.1111/acel.14126 (PMC11113264; doi:10.1111/acel.14126)

Supplementary Figure 1

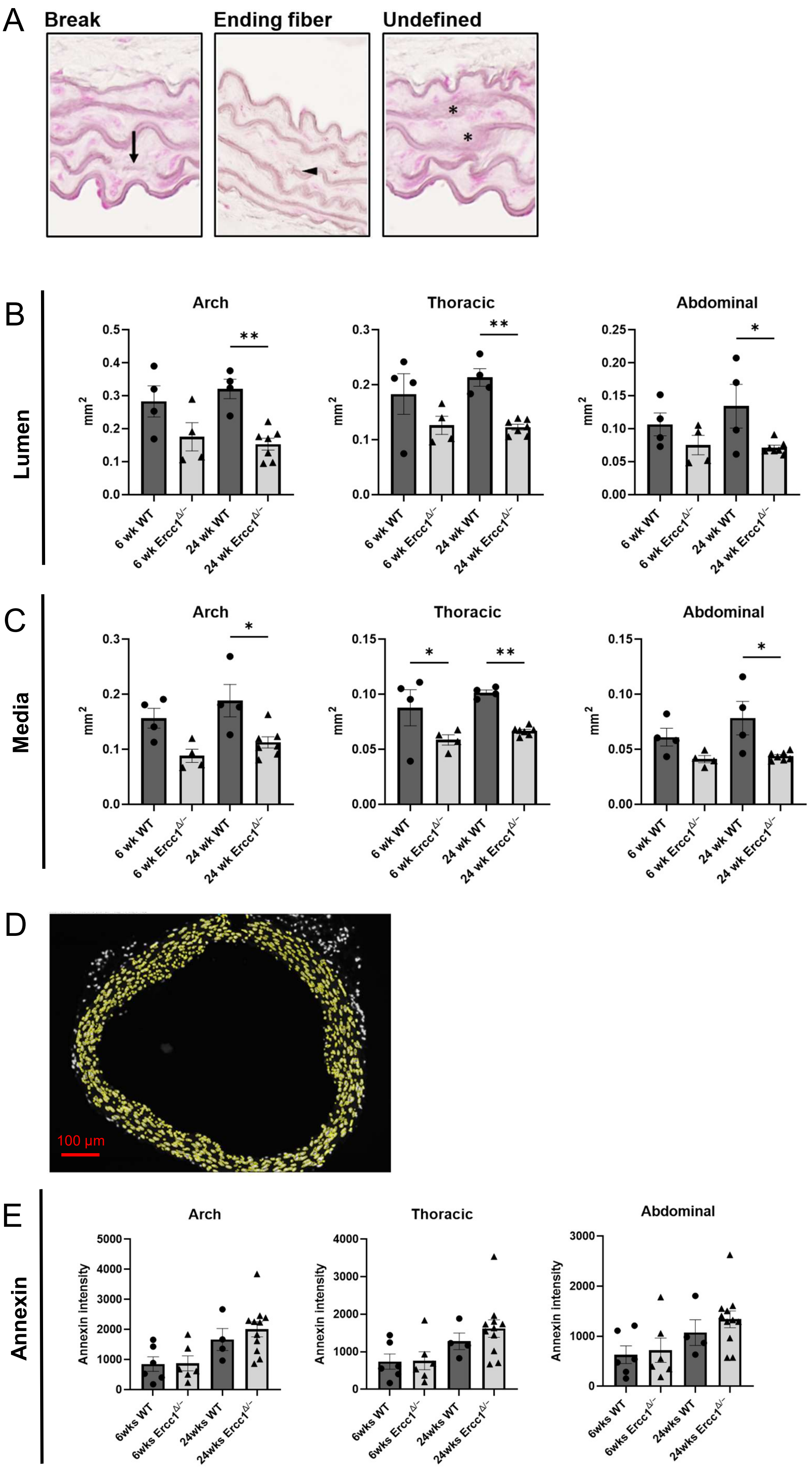

Supplementary Figure 2

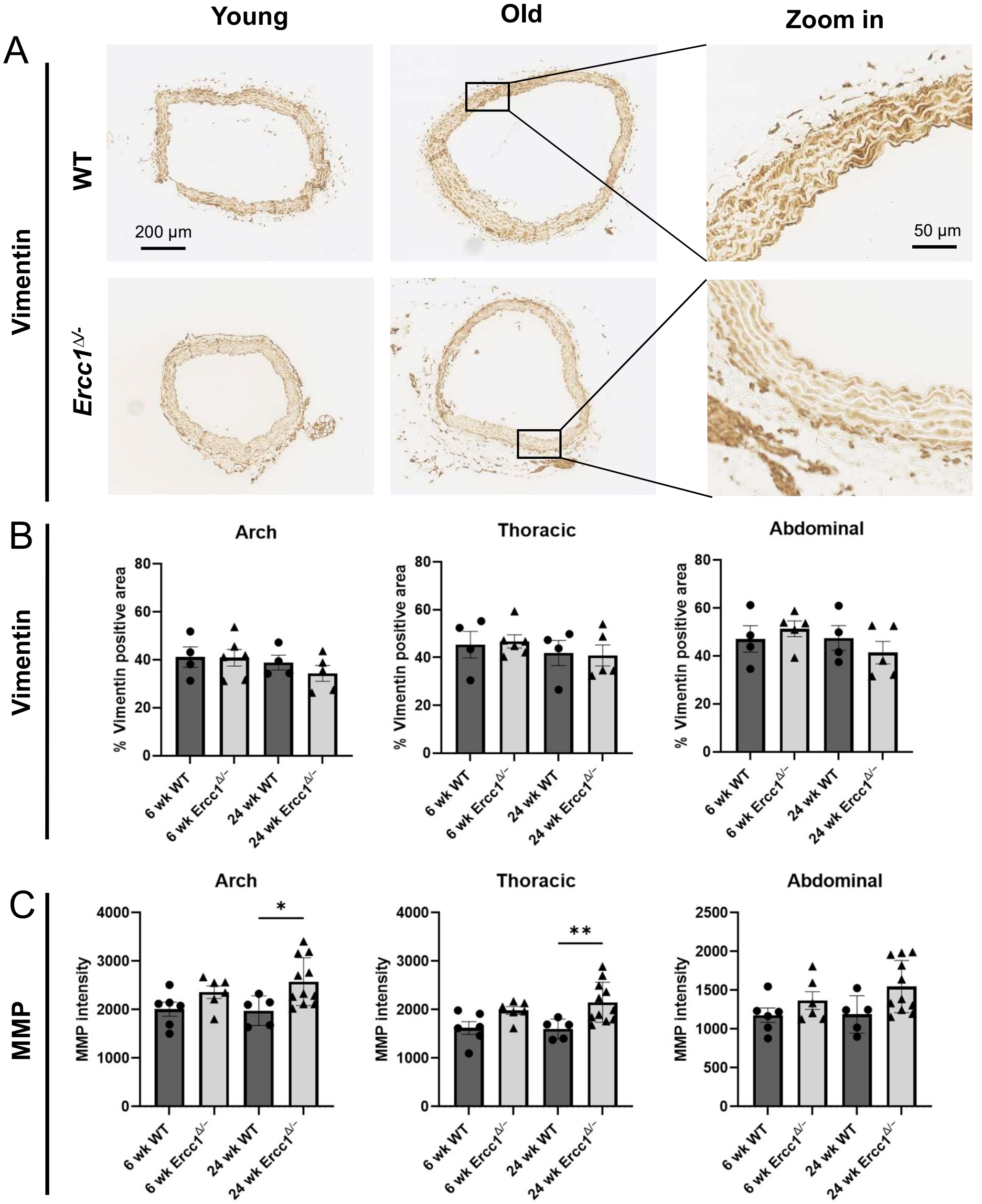

Supplementary Figure 3

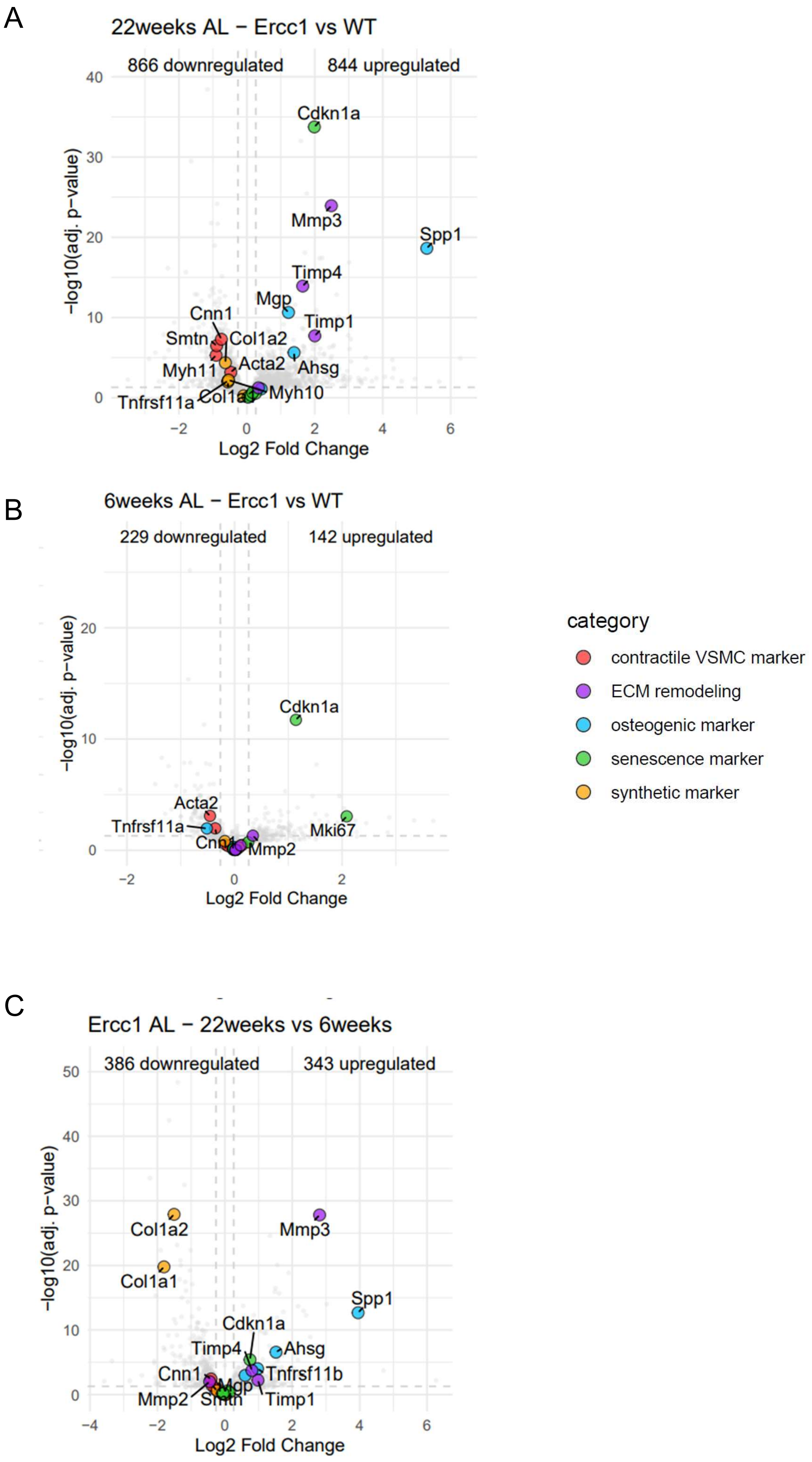

Supplementary Figure 4

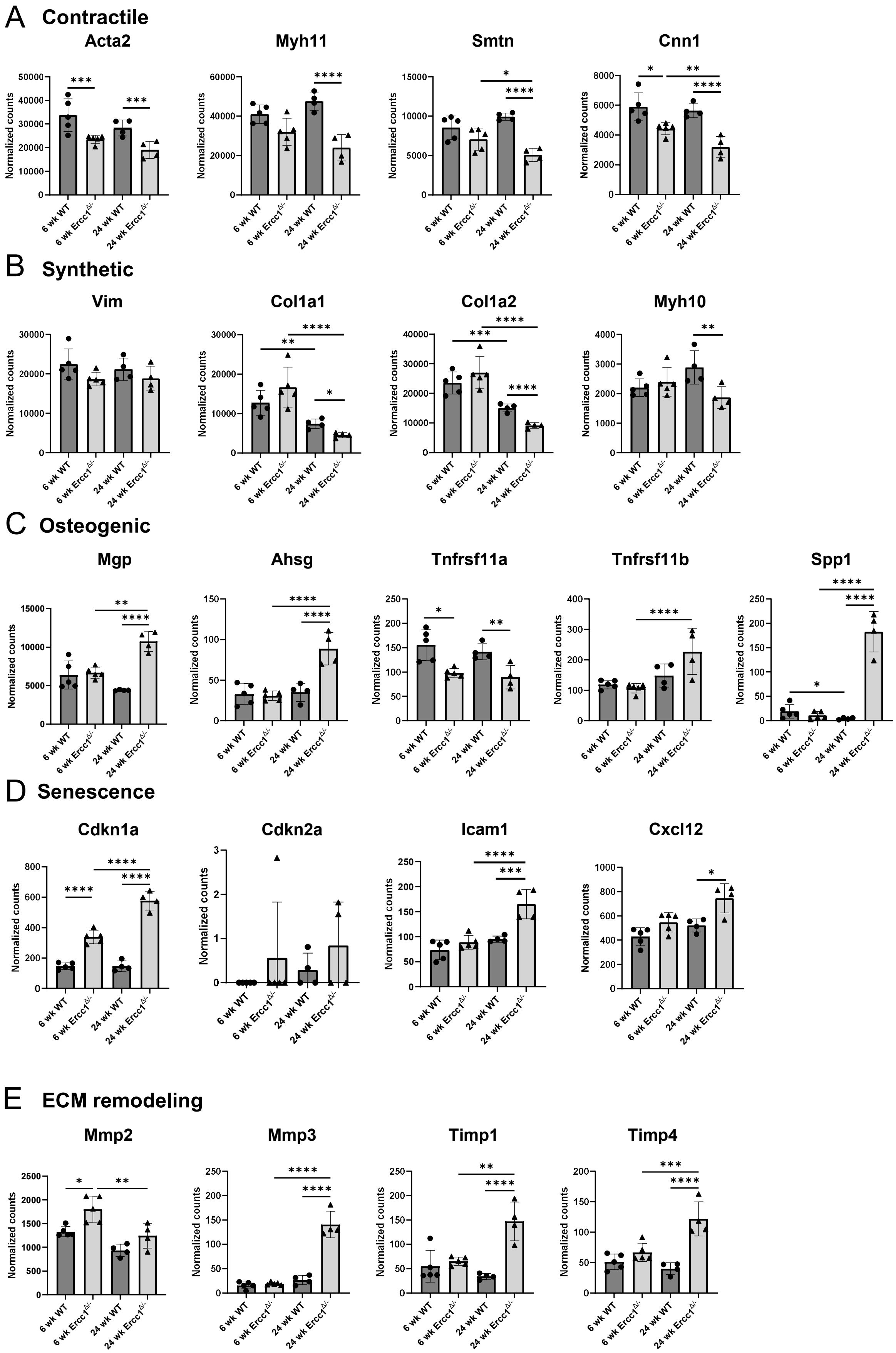

Supplementary Figure 5

A

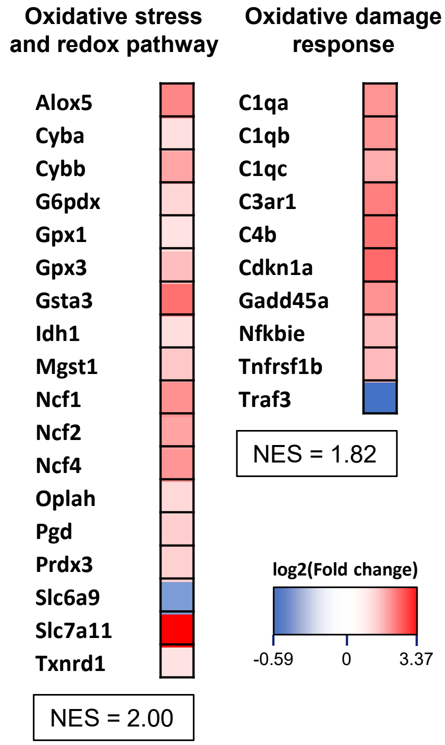

B

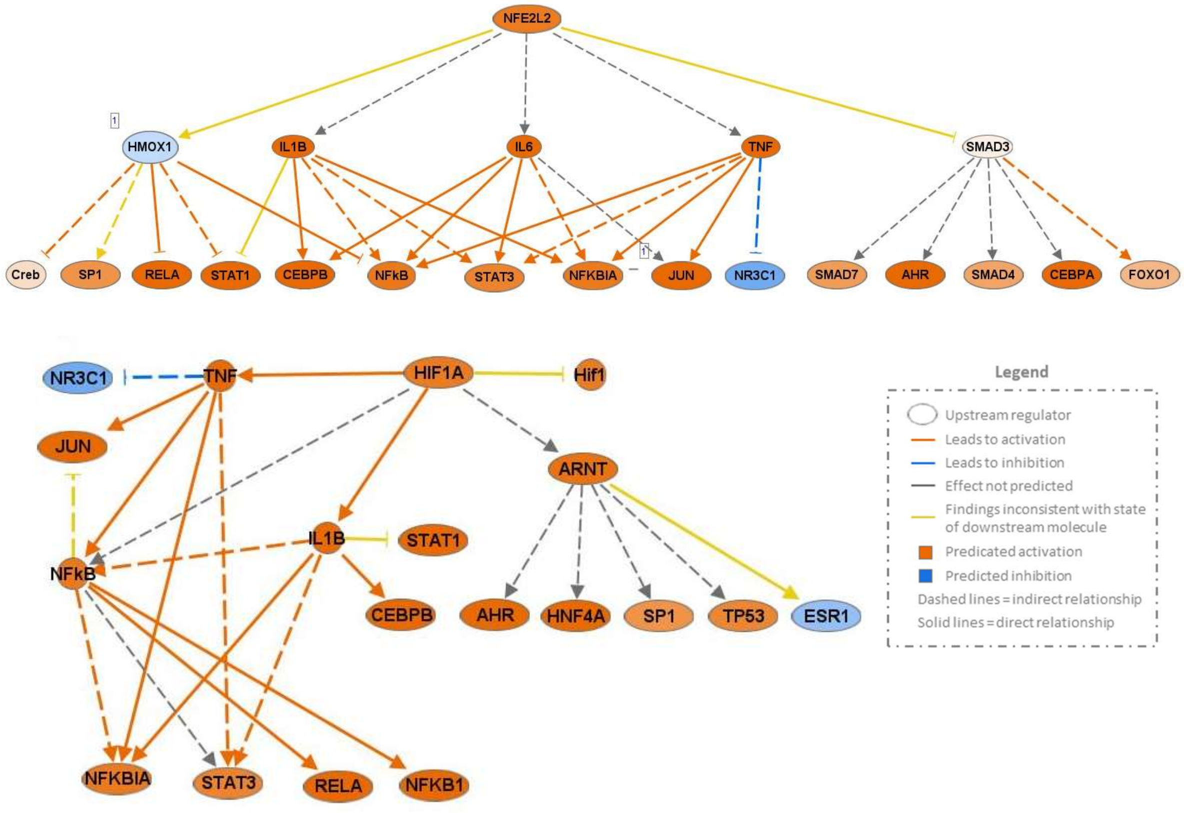

Supplementary Figure 6

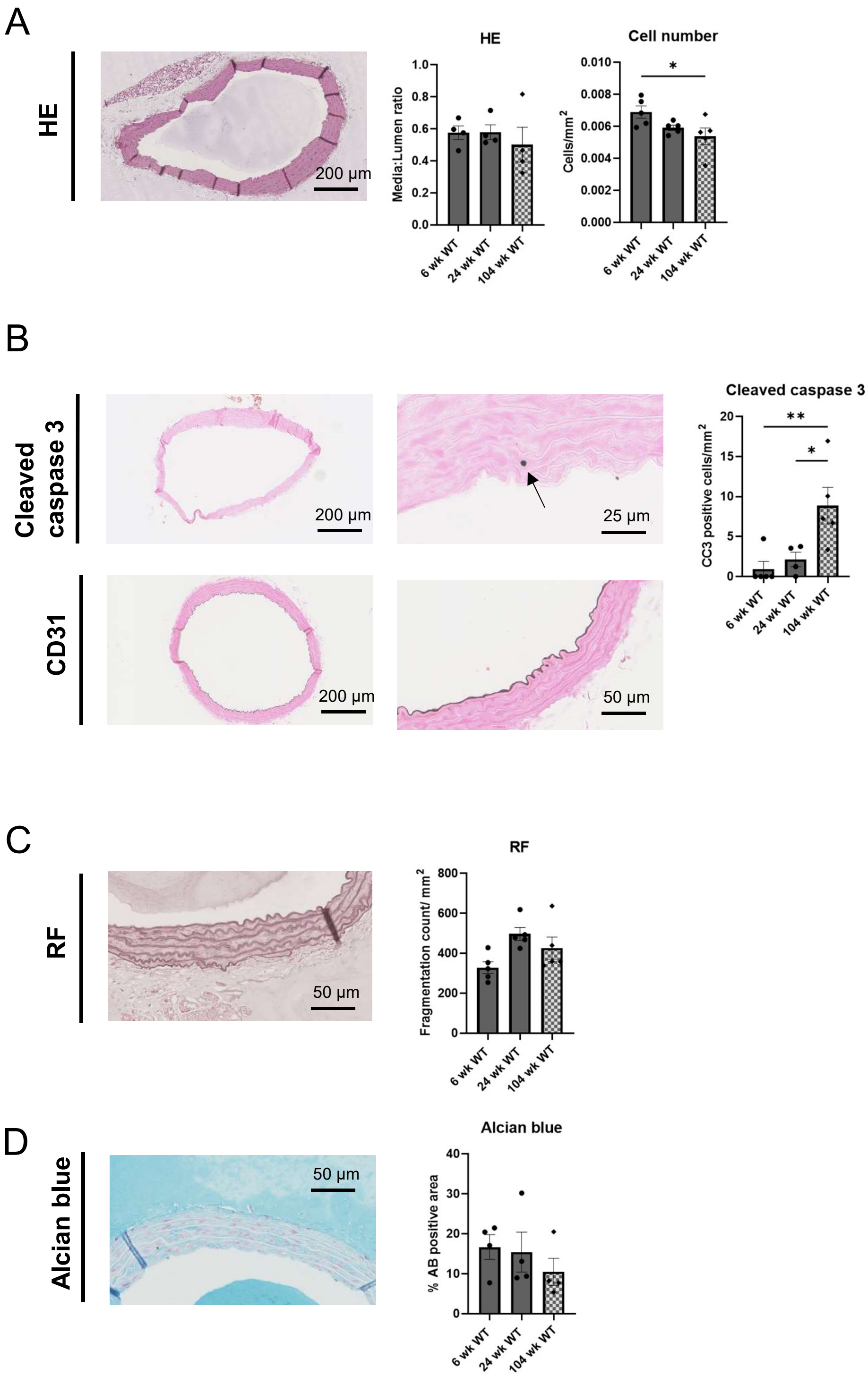

Supplementary Figure 7

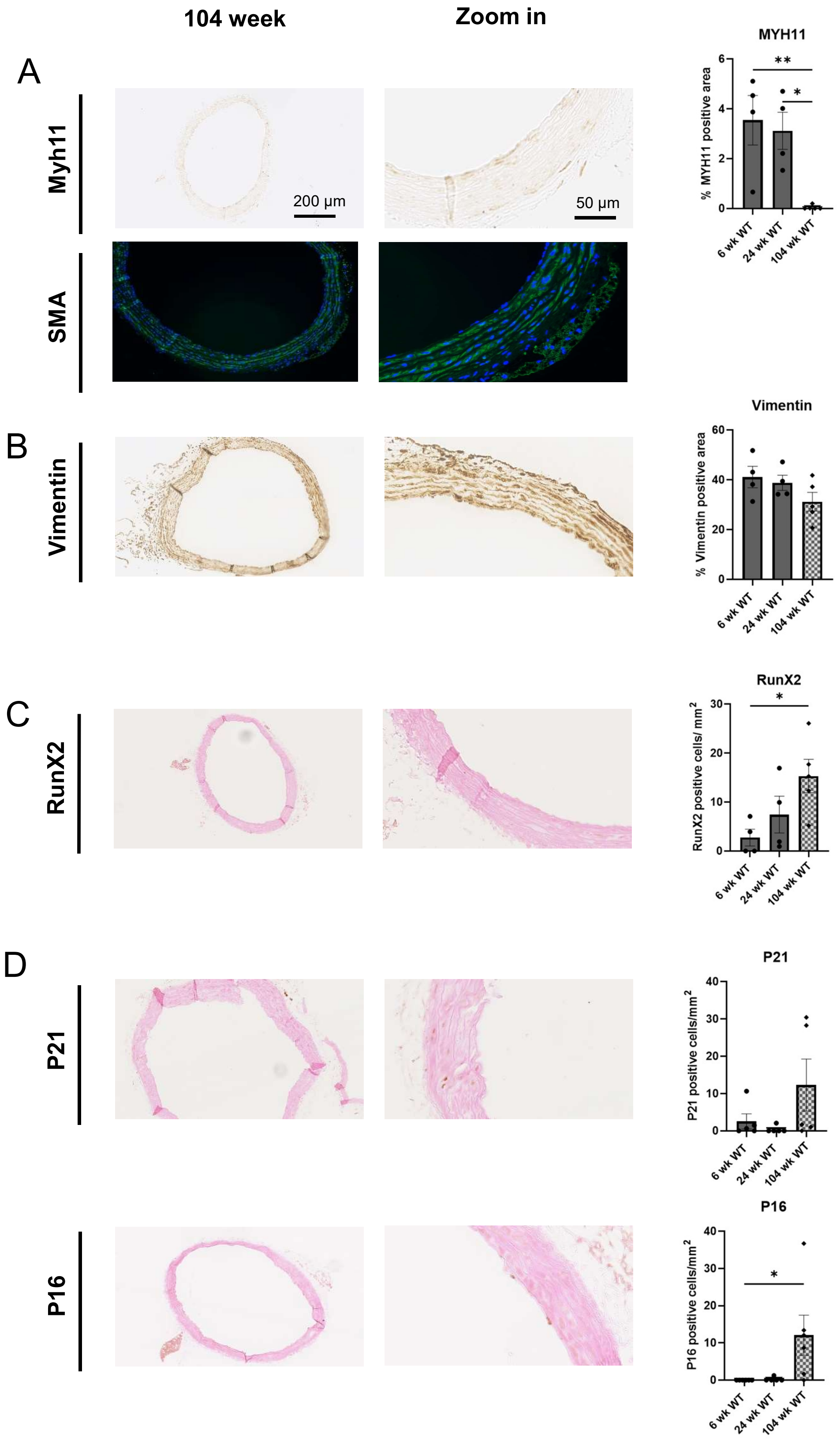

Supplement: Supplementary file 1 — Figures S1–S7. [file ACEL-23-e14126-s001.pdf]
