# Supplementary material for: Ercc1 DNA repair deficiency results in vascular aging characterized by VSMC phenotype switching, ECM remodeling, and an increased stress response
Source: Aging Cell. 2024 Mar 7;23(5):e14126. doi: 10.1111/acel.14126 (PMC11113264; doi:10.1111/acel.14126)
Supplement: Supplementary file 2 — Tables S1–S2. [file ACEL-23-e14126-s002.docx]

**Supplementary:**

**Table 1: Information on mice used for each experiment.**

| **Staining** | **6 wk WT** | **6 wk ERCC1^∆/-^** | **24 wk WT** | **24 wk ERCC1^∆/-^** | **104 wk WT** |
| --- | --- | --- | --- | --- | --- |
| **Weight** | 22,88gr | 15,5gr | 37,06gr | 13,62gr |  |
| **Aortic length** |  |  | 4M/0F | 3M/0F |  |
| **HE** | 2M/2F | 6M/1F | 1M/3F | 3M/4F | 2M/2F |
| **Cell number** | 3M/2F | 6M/0F | 2M/3F | 1M/4F | 2M/3F |
| **Annexin** | 3M/3F | 3M/3F | 2M/2F | 5M/6F |  |
| **Cleaved caspase 3** | 3M/2F | 4M/1F | 2M/2F | 1M/4F | 2M/2F |
| **CD31** | 2M/1F | 3M/0F | 2M/1F | 1M/2F | 1M/2F |
| **MYH11** | 3M/3F | 5M/1F | 2M/3F | 2M/4F | 3M/3F |
| **SMA** | 3M/2F | 6M/0F | 2M/3F | 1M/4F | 2M/3F |
| **RunX2** | 2M/2F | 5M/0F | 1M/3F | 3M/2F | 2M/3F |
| **P21** | 3M/2F | 4M/1F | 2M/3F | 2M/3F | 2M/3F |
| **P16** | 3M/3F | 5M/0F | 2M/3F | 1M/4F | 3M/3F |
| **MMP** | 3M/3F | 3M/3F | 2M/2F | 5M/6F |  |
| **RF** | 3M/2F | 4M/1F | 2M/3F | 2M/3F | 3M/2F |
| **Alcian blue** | 2M/2F | 3M/1F | 1M/3F | 2M/2F | 2M/2F |
| **PDGF** |  |  | 1M/2F | 1M/2F |  |
| **Vimentin** | 2M/2F | 6M/0F | 2M/2F | 2M/3F | 3M/2F |
| **RNA seq** | 3M/2F | 3M/2F | 3M/2F | 2M/2F |  |
| **MOVAT** | 0M/3F | 0M/3F | 2M/3F | 1M/3F |  |

**Table 2: Antibody conditions used.**

| **Protein** | **Antibody ID** | **Dilution** | **Antigen buffer** | **Incubation time** |
| --- | --- | --- | --- | --- |
| **SMA (IF)** | **Ab7817** | **1:500** | **Citrate** | **-** |
| **MYH11** | **Ab224804** | **1:200** | **Citrate** | **4 min** |
| **Vimentin** | **Ab92547** | **1:200** | **Citrate** | **1 min** |
| **RUNX2** | **Ab192256** | **1:100** | **Citrate** | **10 min** |
| **P21** | **Ab107099** | **1:100** | **EDTA** | **4 min** |
| **P16** | **Ab211542** | **1:200** | **Citrate** | **4 min** |
| **Cleaved caspase 3** | **9661S** | **1:100** | **Citrate** | **10 min** |
| **CD31** | **Ab28364** | **1:100** | **EDTA** | **3 min** |

**Supplementary Figure 1. Structural changes in the aorta**. A) Example images to illustrate the quantification of elastin fragmentation. Three types of fragmentation were defined before quantification. Arrows indicate elastin breaks, arrowheads indicate ending fibers and asterisks indicate undefined elastin disarray. All fragmentation types were included in the quantification. B,C) Quantification of the HE staining, indicating a significantly smaller lumen (B) and media (C) size in the old *Ercc1^Δ/−^* aortas compared to wildtype littermates . D) Example image to illustrate cell selection for cell number quantification. E) Analysis of Annexin per region, indicating no significant changes. Sample sizes: HE n=4-7. Results are represented as mean +/− SEM, * p < 0.05, ** p < 0.01. Scale bar= 100µm

**Supplementary Figure 2. No changes in vimentin in *Ercc1^Δ/−^* VSMCs**. A) Representative images of the *Ercc1^Δ/−^* and wildtype aortic arch for Vimentin staining. B) Quantification of the vimentin staining showed no significant changes in the *Ercc1^Δ/−^* aortas compared to wildtype littermates. C) Analysis of MMP per region, indicating a significant increase of MMP in the 24 week old *Ercc1^Δ/−^* compared to the WT. Sample sizes : Vimentin n=4-6. Results are represented as mean +/− SEM. Scale bar= 200µm.

**Supplementary Figure 3. Volcano plots of RNA sequencing data.** Genes are highlighted that belong to 5 different categories: contractile VSMC markers (red), ECM remodelling (purple), osteogenic markers (blue), senescence markers (green) and synthetic markers (yellow). Genes from these categories that are significantly changed are named in the volcano plot (-log10(adjusted p-value > 0.05)>1.3, |log2FC|>(1.2)). A) Volcano plot of DESeq2 analysis of 24 week old *Ercc1^Δ/−^* vs 24 week old WT aorta. The following markers of the highlighted categories were significantly up or down regulated: Acta2, Myh11, Smtn, Cnn1, Col1a1, Col1a2, Myh10, Mgp, Ahsg, Tnfrsf11a, Spp1, Cdkn1A, Mmp3, Timp1 and Timp4. B) Volcano plot of DESeq2 analysis of 6 week old *Ercc1^Δ/−^* vs 6 week old WT aorta, following markers were significantly up or down regulated: Acta2, Cnn1, Tnfrsf11a, Cdkn1a, Mki67 and Mmp2. C) Volcano plot of DESeq2 analysis of 24 week old *Ercc1^Δ/−^* vs 6 week *Ercc1^Δ/−^* aorta, following markers were significantly up or down regulated: Smtn, Cnn1, Col1a1, Col1a2, Mgp, Ahsg, Spp1, Tnfrsf11b, Spp1, Cdkn1A, Mmp2, Mmp3, Timp1 and Timp4.

**Supplementary Figure 4. Normalized counts RNA sequencing data.** Normalized counts (using DESeq2’s median of ratios) of marker genes for 5 different categories: contractile VSMC markers, ECM remodeling osteogenic markers, senescence markers and synthetic markers. A) Normalized counts of contractile markers, showing significant decrease in 24 week old *Ercc1^Δ/−^*aortas compared to the WT littermates. B) Normalized counts of synthetic markers, showing significantly decreased expression of *Col1a1, Col1a2* and *Myh10* in 24 week old *Ercc1^Δ/−^*aortas compared to WT littermates. C) Normalized counts of osteogenic markers, showing significantly increased expression of *Mgp, Ahsg and Tnfrsf11a* in 24 week old *Ercc1^Δ/−^*aortas compared to WT littermates. D) Normalized counts of senescence markers, showing significantly increased expression of *Cdkn1a* in 24 week old *Ercc1^Δ/−^*aortas compared to WT littermates. E) Normalized counts of ECM remodeling markers showing significantly increased expression of *Mmp2, Mmp3, Timp1 and Timp4* in 24 week old *Ercc1^Δ/−^*aortas compared to WT littermates. F) Normalized counts of SASP factors, showing significantly increased expression of *Icam1* and *Cxcl12* in 24 week old *Ercc1^Δ/−^* aortas compared to WT littermates.

**Supplementary Figure5. Oxidative stress pathway analysis RNA sequencing data.**

A) Gene Set Enrichment Analysis of genes from the 24 week old *Ercc1^Δ/−^* vs 24 week old WT comparison revealed enrichment of the Canonical Pathways *“Oxidative stress and redox pathway”* and *“Oxidative damage response”* with a NES score of 2.16 and 2.06 respectively. The genes that are significantly changed in these pathways are mostly upregulated. B) Mechanistic network created by IPA of upstream regulators NFE2L2 and HIF1A, revealing predicted activation of these transcription factors and most of their associated upstream regulators.

**Supplementary Figure 6. Structural changes in the 104 week old aorta**. A) Quantification of the HE staining and cell number, indicating no changes media:lumen ratio, however, a significant loss of cells in the 104 week old WT. B) Representative images of aortic arch of cleaved caspase 3 and CD31, indicating a significant increase in apoptotic cells in the 104 week old aorta, but no interruption in the endothelial layer. C) Representative images of the resorcin fuchsin (RF) staining used to visualize elastin, showing no difference in the 104 week old WT. D) Representative images of the alcian blue (AB) staining, showing no difference between 104 week old and younger WT mice. Sample sizes: HE n=4, Cell number N=5, Cleaved caspase 3 N=4-5, CD31 N=3, RF N=4, AB N=4. Results are represented as mean +/− SEM, * p < 0.05, ** p < 0.01. Scale bar= 25-200µm.

**Supplementary Figure 7. Cellular changes in the 104 week old aorta**. A) Representative images of contractile markers MYH11 and SMA, indicating a significant decrease of MYH11 and SMA at 104 weeks compared to younger WT. B) Representative images of vimentin staining, indicating no changes in vimentin signal in the 104 week old mice compared to younger mice. C) Representative images of RunX2, showed a significant increase of RunX2 in 104 week old WT mice, compared to young WT mice. D) Representative images of P21, indicating a non-significant increase of P21 in the 104 week old WT. E) Representative images of P16 staining, showing a significant increase of P16 in the 104 week old WT. Sample sizes: MYH11 N=5-6, SMA N=5, Vimentin N=4-5, RunX2 N=4-5, P21 N=5, P16 N=5-6. Results are represented as mean +/− SEM, * p < 0.05. Scale bar= 50-200µm.
